# Critical thinking pedagogical practices in medical education: a systematic review

**DOI:** 10.3389/fmed.2024.1358444

**Published:** 2024-06-14

**Authors:** Beatriz Araújo, Sandra F. Gomes, Laura Ribeiro

**Affiliations:** ^1^Department of Public Health and Forensic Sciences, and Medical Education, Medical Education Unit, Faculty of Medicine of the University of Porto, Porto, Portugal; ^2^i3S - Instituto de Investigação e Inovação em Saúde, Universidade do Porto, Porto, Portugal

**Keywords:** critical thinking, clinical reasoning, skills, dispositions, medical education, systematic review

## Abstract

**Introduction:**

The development of critical thinking (CT) has been a universal goal in higher education. A systematic review of the literature was conducted to evaluate the effectiveness of currently used pedagogical practices to foster CT/ clinical reasoning (CR)/ clinical judgment (CJ) skills and/or dispositions in undergraduate medical students.

**Methods:**

PubMed, Web of Science and Scopus databases were searched from January 2010 to April 2021 with a predefined Boolean expression.

**Results:**

Of the 3221 articles originally identified, 33 articles were included by using PICOS methodology. From these, 21 (64%) reported CR pedagogical practices and 12 (36%) CT pedagogical practices.

**Discussion:**

Overall, pedagogical practices such as cognitive/visual representation, simulation, literature exposure, test-enhancing and team-based learning, clinical case discussion, error-based learning, game-based learning seem to enhance CT/CR skills and/or dispositions. Further research is required to identify the optimal timing, duration and modality of pedagogical interventions for effectively foster CT/CR in medical education.

## 1 Introduction

Due to demographic and disease pattern changes along with patient’s needs and aspirations, healthcare professionals are required to develop new skills such as creativity (1), leadership, teamwork, empathy, and communication skills (2), in order to provide high-quality, safe, and effective patient care (3). Physicians must be prepared to deal with all types of environments and make decisions in situations of crisis and epidemics (4). Therefore, skills for managing people-centered care, managing complex tasks, and creating a positive work culture are needed (5). Being aware of the importance of the development of a set of skills beyond knowledge acquisition in 2017, the United Nations Education, Scientific and Cultural Organization (6) suggested the development of critical thinking (CT) as a learning outcome, which is defined as “the ability to question norms, practices, and opinions; to reflect on own[*sic*] one’s values, perceptions, and actions and to take a position in the sustainability discourse” (7). In fact, CT seems to be a key ingredient for commitment to lifelong learning (8) and a deep learning experience, allowing a better understanding and ability to deal with complex concepts and problems (9). It has been positively related to academic achievements (10) and better patients’ assessments, diagnoses, and care in the future (11). Critical thinkers seem to develop a more questioning mind, better critical appraisal abilities, and a positive attitude concerning evidence-based medicine (12). Thus, fostering students to develop CT has been a universal goal “to create better doctors” (13), a desirable outcome that should be developed in an early stage of their training, as skills develop through experience and practice (11, 14).

According to the American Philosophical Association, critical thinking encompasses a broad set of cognitive skills such as interpretation, analysis, evaluation, inference, explanation, self-regulation, and dispositions, including truth-seeking, open-mindedness, analyticity, systematicity, self-confidence, inquisitiveness, and maturity (15). Definitions of CT in medical education tend to emphasize logical or rational thinking—the ability to reason, analyze information, evaluate alternatives, assess arguments and evidence, and reach relevant and appropriate solutions to a problem (16). Moreover, in medicine, CT has also been described and identified nearly as synonymous with “clinical judgment” (CJ), “clinical reasoning” (CR), “diagnostic thinking,” “problem-solving,” or “type 2 thinking” terms involving a mental process used to think through problems and achieve a final decision (11).

Although CT can be taught, both pedagogical and assessment practices are challenging, and there is no consensus on the most effective teaching approach. In part, the instructional methods are challenging due to the different understanding of CT. For instance, Krupat et al. (17) found that 43% of doctors describe CT as a process, 41% as a skill or ability, and 16% as disposition. Therefore, teaching CT remains both a challenge and a necessity in medical education (9).

Our systematic review aims to investigate the effectiveness of the pedagogical practices that are currently used to foster the development of CT/CR/CJ skills and/or dispositions in undergraduate medical students. The specific objectives of this review are as follows: (1) to identify the pedagogical practices currently in use to promote the development of CT/CR/CJ skills and/or dispositions in undergraduate medical students; (2) to identify the tools that are being used to assess CT/CR/CJ skills and/or dispositions in the above conditions; and (3) to investigate the effectiveness of those pedagogical practices, considering the CT/CR/CJ skills and/or dispositions gains, the assessment tools, and the intervention context.

## 2 Methodology

This study followed the Cochrane recommendations (18–22) and the Preferred Reporting Items for Systematic Reviews and Meta-Analysis (PRISMA) Guidelines (23) (Supplementary material—PRISMA Checklist). The protocol for this systematic review is registered on Open Science Framework (OSF): doi: 10.17605/OSF.IO/8PJ26.

Although this systematic review was based on the approach used in a previous study (24), it explored the effectiveness of pedagogical practices used to foster CT/CR/CJ skills and dispositions exclusively in undergraduate medical students while also including other pedagogical outcomes.

### 2.1 Information sources and search strategy

In April 2021, the literature search was conducted in PubMed, Web of Science, and Scopus using the following Boolean expressions: (“Critical Thinking” OR “Clinical Reasoning” OR “Clinical Judgement”) AND (Skill OR Ability OR Disposition OR Attitude) AND (Strategies OR Interventions OR Educat* OR Teach* OR Practice OR Train OR Develop* Analyse* OR Test* OR Evaluate* OR Assess*) AND (Student* OR Undergraduate* OR School OR Faculty OR College OR High* Education OR Universities) AND (Medic*). The filters “Article title, Abstract, and Keywords” and “All fields” were used in Scopus, PubMed, and Web of Science databases. In addition, a time filter from January 2010 to April 2021 was set, and the studies were included according to the eligibility criteria.

### 2.2 Eligibility criteria

Studies were deemed eligible according to the following inclusion and exclusion criteria defined using the PICOS tool (25):

- P (Population) — undergraduate medical students;
- I (Interventions) — pedagogical practices to foster the development of CT/CR/CJ skills and/or dispositions;
- C (Comparison) — not applicable;
- O (Outcomes) — CT/CR/CJ skills and/or dispositions gains;
- S (Study design) — Qualitative, quantitative, and mixed studies.

Articles published from January 2010 to April 2021, referring to CT/CR/CJ pedagogical practices as interventions and undergraduate medical students as its target population, were included. Furthermore, articles with a clear description of the pedagogical practices that were used to foster the development of CT/CR/CJ were also included. For this systematic review, studies with qualitative, quantitative, and mixed-method designs were considered.

Letters, short communications, systematic reviews, reviews, and meta-analysis were excluded. Studies lacking a clear methodological description of the intervention, articles outside the scope of undergraduate medical education or studies with no access to the full text despite the attempt to contact the authors, were also excluded during the screening phase. Although the search terms used were in English, no language restrictions were applied in our research strategy.

### 2.3 Study selection

Studies were screened and selected by two independent reviewers. After duplicate records were removed, first, studies were screened based on the title and then based on the abstract. The remaining records were eligible for full paper reading based on the inclusion and exclusion criteria. Any disagreement was solved by consensus.

### 2.4 Data collection process

Data were collected, organized, and synthesized in tables based on the following: author(s), publication year, country, study design, objectives, sample, pedagogical approach (pedagogical practices, curricular context, subject specificity, regime, subject, length of the intervention, interventional and/or control group, format, instructional support, and feedback), assessment tools (pre-intervention and post-intervention), and main findings (Supplementary material — data collection).

### 2.5 Quality assessment

The included studies underwent a quality assessment by two independent reviewers using the Standard Quality Assessment Criteria for Evaluating Primary Research Papers from a Variety of Fields (26). The studies received scores for their compliance (“yes” = 2, “partial” = 1; and “no” = 0), with each of the 10 criteria for qualitative studies, 14 criteria for quantitative studies, and both (24) criteria for mixed studies. For each study, a sum score was calculated by adding the scores for these criteria and dividing them by the total possible score (26).

### 2.6 Data management

To synthesize the information and enable comparison between pedagogical practices, some studies were characterized and grouped according to the characteristics of the pedagogical approach (i.e., cognitive/visual problem representation when a cognitive knowledge organization strategy was used such as mind map, conceptual mapping, illness script, or case vignettes; simulation when a low-fidelity or high-fidelity patient simulation was used; literature exposure when students were exposed and instructed to reflect based on books, literary excerpts, or papers).

Then, pedagogical practices were also divided into CT, CR, or CJ. In studies where multiple pedagogical practices were mentioned (e.g., debates during problem-based learning), only the most prominent studies focusing on the development of CT/CR/CJ skills and/or dispositions were considered to characterize the intervention except for the innovative curriculums that purposefully combine different pedagogical practices.

The curricular context of the pedagogical approach was categorized as curricular (when the CT/CR/CJ pedagogical practices are implemented during the formal curriculum within the context of a specific year and subject content as part of the objectives of a given curricular unit) or extracurricular (when the CT/CR/CJ pedagogical practices are implemented during an elective course or workshop with a certain group of students regardless of the objectives of any curricular unit of the formal curriculum). According to Ennis (27), subject specificity was characterized as follows: general—an approach that attempts to teach CT/CR/CJ abilities and dispositions regardless of the subject content; infusive—an approach where students are encouraged to think critically in the subject (subject-related), in which general CT/CR/CJ principles are made explicit to the students; immersive—an approach where students are encouraged to think critically in the subject (subject-related), in which general CT/CR/CJ principles are not made explicit to the students; or mixed—an approach that combines the general approach with one of the other two, infusive or immersive.

Additionally, the analysis also considered the length of the intervention, the learning regime (face-to-face vs. e-learning), the work format (individual vs. collaborative), the presence or absence of a control group, the number of group interventions, the presence or absence of instructional support (contextualization, facilitators/tutors ‘guidance, or guidelines), or feedback during or at the end of the interventions.

To simplify the comparison between CT/CR/CJ pedagogical practices and learning outcomes, the assessment approach was classified according to the tool standard (standardized or non-standardized), the domain specificity (general, health sciences, or medical domain), and the assessment tool specificity to evaluate CT/CR/CJ skills and/or dispositions (tests or rubrics, knowledge tests, self-assessment surveys or questionnaires, and focus groups session).

Since some studies used more than one assessment tool, only the most effective in evaluating CT/CR/CJ pedagogical practices was used to describe the assessment approach in the following order:

1. Domain-specific standardized tests—any quantitative assessment tools used to measure CT/CR/CJ skills and/or dispositions specifically in the health science domain [e.g., The Health Science Reasoning Test (HSRT); Yoon’s Critical Thinking Disposition Instrument (YCTDI); Critical Thinking Disposition Assessment (CTDA); Objective Structured Clinical Examination (OSCE); and the Diagnostic Thinking Inventory (DTI)];
2. General standardized tests—any quantitative assessment tools used to measure CT/CR/CJ skills and/or dispositions with no specific domain [e.g., The California Critical Thinking Skills Test (CCTST); the Ennis–Weir Critical Thinking Essay Test; and the California Critical Thinking Disposition Inventory (CCTDI)];
3. Domain-specific non-standardized tests or rubrics—a quantitative assessment tool previously developed or adapted specifically to assess CT/CR/CJ skills and/or dispositions or students’ performance in CT/CR/CJ regarding the domain and the context of the intervention [e.g., Script Concordance Test (SCT), Key Feature Problem Examination (KFPE); Critical Thinking Skills Rating Instrument (CTSRI) — rubric, the Clinical reasoning performance — assessed with 3 knowledge tests as follows: (1) conceptual knowledge test with multiple choice questions, (2) strategic knowledge test with key feature questions, and (3) conditional knowledge with problem solving tests, clinical reasoning test, or problem solving tests assessed with rubrics];
4. Domain-specific non-standardized knowledge tests, self-assessment surveys, or questionnaires—a quantitative non-standardized or self-reported assessment tool previously developed to assess the learning experience as student knowledge retention, self-perception, or satisfaction with the pedagogical approach;
5. Domain-specific non-standardized focus group sessions—a qualitative non-standardized self-report assessment tool used to assess the learning experience such as the perception or satisfaction of the students with the pedagogical approach.

Considering the most significant assessment tool for the evaluation of the CT/CR/CJ pedagogical practices, the learning outcomes were classified as follows:

- CT/CR/CJ general gain (++) when a statistically significant gain in terms of a general set of CT/CR/CJ skills or dispositions was verified (based on the general score of domain-specific standardized tests, general standardized tests, or domain-specific non-standardized tests or rubrics);
- CT/CR/CJ specific gain (+) when a statistically significant gain was reported for a specific CT/CR/CJ skill or disposition (based on the individual score related to a specific item of domain-specific standardized tests, general standardized tests, or domain-specific non-standardized tests or rubrics);
- CT/CR/CJ no gain (−−) when no statistic gain in terms of a general set of CT/CR/CJ skills or dispositions was verified (based on the general score of domain-specific standardized tests, general standardized tests, domain-specific, or non-standardized tests or rubrics);
- Other gains (+?) as knowledge, satisfaction, or perception of the development of CT/CR/CJ skills and/or dispositions when a statistically significant gain or a qualitative gain was verified regarding the student learning experiences (based on the general score of domain-specific non-standardized tests, surveys or questionnaires, or a qualitative result of domain-specific focus group sessions).
- No other gains (−?) as knowledge, satisfaction, or perception of the development of CT/CR/CJ skills and/or dispositions when no statistically significant gain or a qualitative gain was verified regarding the student learning experiences (based on the general score of domain-specific non-standardized tests, surveys or questionnaires, or a qualitative result of domain-specific focus group sessions).

## 3 Results

### 3.1 Study selection

**Figure 1:**
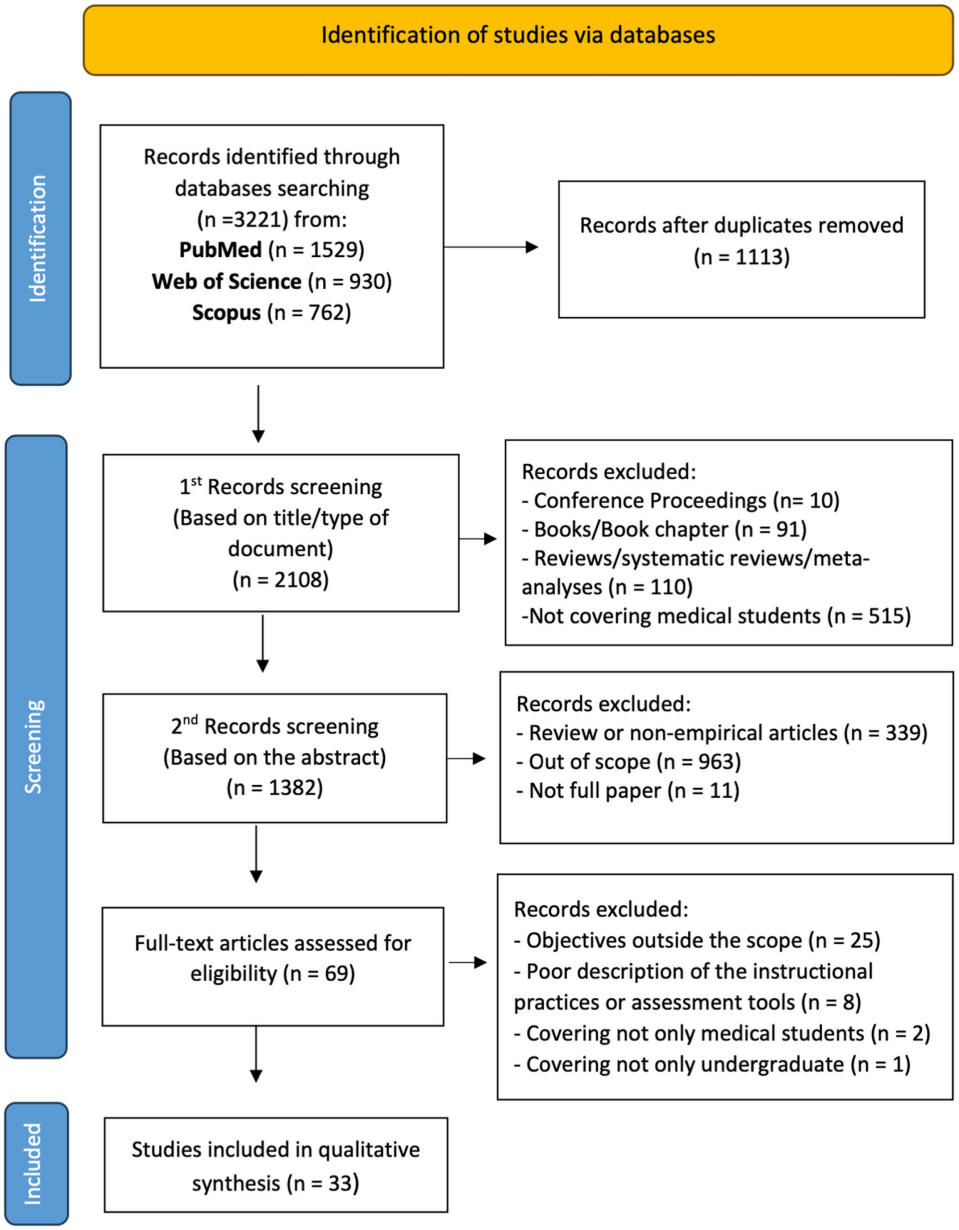
Flow PRISMA diagram of the included studies.

A total of 3,221 articles were identified through database screening and were subsequently subjected to a stepwise filtering process (Figure 1). After duplicate records were removed, the remaining 2,108 studies were screened in two phases. In the first phase, 726 studies were excluded based on the title and document type. In the second phase, 1,314 studies were excluded based on the abstract by applying the exclusion criteria. From the remaining 69 eligible records for full paper reading, 35 were excluded for not meeting the inclusion criteria. The remaining 33 studies were included in this systematic review.

### 3.2 Quality assessment

The results of the quality assessment are summarized in the Quality Assessment Table (Supplementary material). The quality rating ranged from 0.62 to 0.95 with a mean of 0.75. Lower ratings were due to a poor description of the sampling strategy and a lack of evidence of both verification procedures and reflexibility in qualitative and mixed studies. In quantitative and mixed studies, lower ratings were due to poor description and appropriateness of the sampling, incomplete baseline/demographic data, and lack of confounding assessment.

### 3.3 Population

The included studies were markedly different in sample size, the smallest comprising 10 first-year medical students (28) and the largest comprising 214 third-year medical students (29).

The CT/CR/CJ pedagogical practices were applied to students attending the first 5 years of the medical course, with most studies covering the fourth year (*n* = 8) followed by the first (*n* = 6), second (*n* = 4), and third year (*n* = 4) (Tables 1, 2). In addition, of the 33 studies, 8 (40, 41, 46–49, 56, 58) studies recruited students from different academic years. Additionally, 61% of the studies did not mention the students’ age and 30% did not mention their gender.

**Table 1:** Characteristics of the studies employing critical thinking pedagogical practices.

Author, data	Sample	Pedagogical approach						Assessment tool	Outcomes
		Pedagogical practice		Specificity	Subject/topic	Length	Regime		
Archila (30)	*n* = 91 62% (56) F 1st y	**Literature exposure**	Argument evaluation -drama-based CT classroom scenarios	Immersive	ethics, social responsibility, and scientific work	1 session (60 min)	Individual	Domain-specific non-standardized knowledge tests, self-assessment surveys or questionnaires	Other gains (+?)
Kim (31)	*n* = 51 25% (13) F 2nd y		Read/watch the material + group discussion sessions + writing a critical essay	Immersive	Social sciences and humanities	15 weeks	Collaborative (groups of 10–11)	Domain-specific standardized tests	CT/CR/CJ Specific gain (+)
Liao and Wang (32)	*n* = 82 –		Gender perspective into literature + reflection + e-discussion	Immersive	Gender literature studies	15 weeks (2xs/week)	Individual + Collaborative (with group mate)	General standardized tests	CT/CR/CJ Specific gains (+)
D'Antoni et al (33)	*n* = 131 52% (68) F 1st y	**Cognitive/visual representation**	Mind map *vs* standard notetaking	Mixed	Cacti and other succulent plants	1 session (205 min)	Individual	Domain-specific standardized tests	CT/CR/CJ No gain (−)
Bixler et al. (14)	*n* = 33 – 4th y		Concept mapping in small groups	Immersive	Pediatric topics	4 sessions; (1 h/session)	Collaborative (groups of 4–6)	General standardized tests	CT/CR/CJ No gain (−)
Mumtaz and Latif (34)	*n* = 182 100% F 2nd y	**Debate** (during PBL)		Immersive	Areas of controversy in medicine	1 year/2 semesters 6–7 sessions	Collaborative (groups of 10–13)	Domain-specific non-standardized knowledge tests, self-assessment surveys or questionnaires	Other gains (+?)
Nguyen et al. (35)	*n* = 120 – 1st y	**Simulation—**high-fidelity patient simulations –manikins		Immersive	Physiology	17 weeks (55–60 min each session)	Collaborative (groups of 6)	Domain-specific non-standardized tests or rubrics	CT/CR/CJ General gain (++)
Banerjee et al. (36)	*n* = 54 – 1st y	Mentored**journal clubs**: 6D-approach		Immersive	Molecular biology and principles of genetics	15 weeks (7 sessions)	Collaborative (groups of 3–4)	Domain-specific non-standardized knowledge tests, self-assessment surveys or questionnaires	Other gains (+?)
Sahoo and Mohammed (9)	*n* = 188 56% (105) F 4th y	**Reflective writing**: collaborative research protocol writing		Immersive	Ophthalmology	4 weeks	Collaborative (Small groups)	Domain-specific non-standardized knowledge tests, self-assessment surveys or questionnaires	Other gains (+?)
Ghiam et al. (37)	*n* = 100 – 2nd y	**Dialogue narrative approach:** storytelling format + question-answer conversational style at regular intervals and flipped classroom		Immersive	Thyroid physiology	1 session (50 min)	Individual	Domain-specific non-standardized focus group sessions	Other gains (+?)
McClintic et al. (29)	*n* = 214 44% (94) F 3th y	**Innovative curriculum:** entrustable professional activities ^(a)^		Immersive	Surgical clerkship	8 sessions	Individual + Collaborative (small groups)	Domain-specific standardized tests	CT/CR/CJ general gains (++)
Taghinezhad and Riasati (38)	*n* = 140 Both genders	**Explicit CT instructions** ^(b)^		Infusive	Parking problem in a small town/CT concepts	1 semester; 15 weeks (3 h each)	Individual	General standardized tests	CT/CR/CJ Specific gains (+)

PBL-problem based learning; (a) deliberated-practice structured orientation + small group sessions + online quizzes + extensive didactics + team-based learning and simulation exercises + clinical portfolio; (b) providing CT explicit instruction, teaching students how to make use of those techniques to synthesize, analyze, and evaluate information, presenting support materials in CT classrooms (including leaflets and models) of the instructional techniques, leading Socratic discussions based on the elements and criteria suggested in the instructional techniques, assigning classroom activities and giving them adequate time to practice each skill, using both oral and written techniques, and assessing students’ performance.

**Table 2:** Characteristics of the studies employing Clinical Reasoning pedagogical practices.

Author, data	Sample	Pedagogical approach						Assessment tool	Outcomes
		Pedagogical practice		Specificity	Subject/topic	Length	Regime		
Lee et al. (39)	*n* = 53 51% (33) F 4th y	**Cognitive /visual representation**	Problem representation + illness script – web-based CR problems	Infusive	Two scenarios: 1) an elderly man with a persistent cough; 2) a middle-aged woman with an acute swollen and painful left leg	1 session (3 h)	Collaborative (small groups)	Domain-specific standardized tests	CT/CR/CJ no gain (−)
Wu et al. (40)	*n* = 29 66% (19) F 4th y (19) 3rd and 5th y (10)		Computer-based argument mapping + concept mapping	Immersive	Kidney disease	4 weeks (5 h/week)	Individual	Domain-specific non-standardized tests or rubrics	CT/CR/CJ General gain (++)
Si et al. (41)	*n* = 95 41.1% (39) F 1st y (44) and 2nd y (51)		Argumentation with the concept map method during PBL – according to Toulmin’s model of argumentation	Immersive	Clinical cases	3 sessions (2 h each) 3 weeks	Collaborative (groups of 7–8)	Domain-specific non-standardized tests or rubrics	CT/CR/CJ General gain (++)
Kumar et al. (42)	*n* = 150 – 1st y		clinical-anatomical case vignettes for analyzing clinical cases	Immersive	Varicose veins and thyroid goiter	2 sessions (1 h each)	Collaborative (groups of 2)	Domain-specific non-standardized tests or rubrics	CT/CR/CJ General gains (++)
Moghadami et al. (43)	*n* = 100 53% (53) F 4th y		Illness script - > small group discussion (think aloud) - > debriefing	Immersive	Cirrhosis / CHF / Nephrotic Syndrome /leg edema	2 sessions (7 h each) 4 weeks	Individual + collaborative (small group discussion + open discussion)	Domain-specific non-standardized tests or rubrics	CT/CR/CJ General gains (++)
Middeke et al. (44)	*n* = 112 56.3% (63) F 5th y	**Game-based learning—**Serious Game (playing EMERGE) *vs* small-group PBL		Immersive	internal medicine (Cardiology, pulmonology nephrology, rheumatology hematology, oncology)	6 weeks 10 sessions (90 min each)	individual *vs* collaborative (groups of 6–8)	Domain-specific non-standardized tests or rubrics	CT/CR/CJ General gain (++)
Chandrasekar et al. (28)	*n* = 10 – 1st y	**Case creation**—“build-a-case” approach *vs* traditional CBL		Immersive	Dilated cardiomyopathy	1 session (3 h)	Collaborative (groups of 5)	Domain-specific non-standardized focus group sessions	Other gain (+?)
Brich et al. (45)	*n* = 122 57.4% (70) F 3rd (92) and 4th (30) y	**Team-based Learning**	(symptom-oriented small-group seminars or sTBL units)	Infusive	Neurology topics (vertigo, acute back pain, first epileptic seizure, and acute altered mental status)	2 weeks	Collaborative (small groups)	Domain-specific non-standardized tests or rubrics	CT/CR/CJ General gain (++)
Jost et al. (46)	*n* = 26 58% (15) F 4th (18) and 5th y (8)		TBL vs. non-TBL	Immersive	(vertigo, acute back pain, first epileptic seizure, and acute altered mental status)	4 sessions (90 min/session)	Collaborative (5–7 students) vs. individual	Domain-specific non-standardized tests or rubrics	CT/CR/CJ General gain (++)
Klein et al. (47)	*n* = 84 67% (56) F clinical semesters	**Error-based learning**: learning from errors in a clinical case-based online learning environment (text vignettes)		Immersive	arterial hypertension	1 session (no time limit)	Individual	Domain-specific non-standardized tests or rubrics	CT/CR/CJ General gain (++)
Schubach et al. (48)	*n* = 56 67% (38) F 4th and 5th y	**Simulation**	VPs + key feature-based instructions on multiple short cases *vs* VPs + systematic instruction on a few long cases	Immersive	Acute abdomen gastrointestinal bleeding	3 sessions (90 min/session) 2 weeks	Individual work - > small group discussion - > moderated group discussion	Domain-specific non-standardized tests or rubrics	CT/CR/CJ No gain (−)
Isaza-Restrepo et al. (49)	*n* = 20 – 1st to 3rd y		Web-based VPs: low-fidelity simulator of clinical cases	Infusive	Abdomen pain of different etiology	16 weeks- 2 sessions per week (2 h per session)	Collaborative (small groups)	Domain-specific non-standardized tests or rubrics	CT/CR/CJ General gain (++)
Mutter et al. (50)	*n* = 96 – 4th y		High-fidelity simulation (patient case scenario with *vs* without manikin)	Immersive	Chest pain	1 session (2 h)	Collaborative (6 students)	Domain-specific non-standardized tests or rubrics	CT/CR/CJ General gains (++)
Watari et al. (51)	*n* = 169 37% (63) F 4th y		VPs (®Body Interact, Portugal)	Infusive	Two scenarios: 1) a 55-year-old male with altered mental status; 2) a 65-year-old male with acute chest pain	1 session (2 h)	Individual	Domain-specific non-standardized tests or rubrics	CT/CR/CJ General gains (++)
Kleinert et al. (52)	*n* = 62 – 3rd year		VPs (ALICE)	Immersive	Esophageal cancer (different tumor stages and different therapeutic options)	-	Collaborative (small groups - <5 MS)	Domain-specific non-standardized tests or rubrics	CT/CR/CJ General gain (++)
Ludwig et al. (53)	*n* = 93 64.5% (60) F 4th y	**Test-enhanced learning**	video-based key feature questions *vs* repeated testing with text-based on key feature questions	Immersive	cardiology, pulmonology, nephrology, rheumatology, hematology, and oncology	10 weeks 1 session per week (45 min)^(a)^	Individual	Domain-specific non-standardized tests or rubrics	CT/CR/CJ General gain (++)
Raupach et al. (54)	*n* = 87 58,6% (51) F 4th year		Computer CBL + augmented case presentation + key feature questions *vs* repeated CBL (long case narratives)	Immersive	Cardiology, pulmonology nephrology, rheumatology hematology, and oncology	10 weeks 1 session per week (45 min)^(a)^	Individual	Domain-specific non-standardized knowledge tests, self-assessment surveys, or questionnaires	CT/CR/CJ General gain (++)
Montaldo Lorca and Herskovic (55)	*n* = 64 – 3rd y	**Clinical case discussion (CCD)**	Prototypical clinical cases (lectures and tutorial sessions with patients *vs* with patients + discussion seminars).	Immersive	Semiology and Internal Medical clerkship cardiac and pulmonary pathology syndromes	6 months	Collaborative (small groups)	Domain-specific non-standardized tests or rubrics	CT/CR/CJ General gain (++)
Weidenbusch et al. (56)	*n* = 90 65.5% (59) F 1st to 4th y		(Live-CCD *vs* Video – CCD *vs* Paper – cases)	Immersive	paresthesia, fever, and respiratory failure, rapidly progressive respiratory failure	3 weekly—5 sessions (90 min each) ^(b)^	collaborative vs. individual	Domain-specific non-standardized tests or rubrics	CT/CR/CJ General gain (++)
Bonifacino et al. (57)	*n* = 67 – 3rd year	**Innovative curriculum:** six interactive online modules – didactic videos, simulated clinical cases, and interactive prompts for open-ended MCQ; and a case-based workshop		Infusive	diagnostic error, cognitive psychology of decision-making, specific CR skills, semantic qualifiers and problem representation, cognitive biases, and heuristics	4 weeks	Individual and collaborative (small groups of 3–4 students + large groups)	Domain-specific non-standardized tests or rubrics	CT/CR/CJ General gain (++)

PBL – problem based learning; CBL – case based learning; TBL – team based learning. VPs – Virtual Patients simulation; CBL – case-based learning; CCD – clinical case discussion; MCQ – multiple choice questions (a) 13th week (exit exam) 9th month (retention test); (b) 3rd week (exit exam), 5th week (retention test).

### 3.4 Pedagogical practices, assessment tools, and learning outcomes

A diversity of pedagogical practices was used to foster the development of CT/CR/CJ skills and/or dispositions in undergraduate medical students (Tables 1, 2). The most frequently used were cognitive/visual representation approaches (8/33, 24.2%) as mind map (33), concept mapping (14, 40, 41), clinical-anatomical case vignettes (42), and illness script (39, 43, 59). In addition, simulation was also frequent (6/33, 18.2%) through virtual patients—low-fidelity patient simulations (48, 49, 51, 52) and manikins—high-fidelity patient simulations (35, 50).

Literature exposure was also reported (3/33, 9.0%) involving drama-based scenarios (30) and literature and film analysis (31) and integrating a gender perspective into literature studies (32).

Other approaches, such as test-enhancing learning (53, 54), team-based learning (46, 58), clinical case discussion (55, 56), case creation, team-based learning (46), game-based learning (44), error-based learning (47), dialog narrative approach (37), reflective writing (9), journal club (36), debate (34), and the explicit CT instruction approach (38), were mentioned in a few studies. Furthermore, some studies (29, 57) employed an innovative curriculum that combines different pedagogical approaches.

Pedagogical practices, such as cognitive/visual representation approaches, mind maps (33), illness-script (39, 43, 59), clinical-anatomy case vignettes (42), literature exposure by integrating the gender perspective into literature studies (32), simulation (50), clinical case discussion (55), team-based learning (46), test-enhancing learning (53, 54), explicit CT instruction approach (38), and the innovative curriculums (29, 57), were compared with the traditional format while case creation was compared with case-based learning (28).

Overall, CT/CR/CJ pedagogical practices were employed in both “curricular” (16/33; 48.5%) and “extracurricular” (17/33; 51.5%) contexts, predominantly with an “immersive” approach (25/33; 75.8%) and in a face-to-face regime (27/33; 78.8%).

The subjects/topics covered by the interventions were quite variable. Overall, as described in Tables 1, 2, they were mainly related to the medical curriculum (31/33; 93.9%), with just a few more associated with medical humanities (4/33; 12.1%) or not directly related to the typical medical curriculum topics (2/33; 6,1%) (33, 38).

Regarding other factors of the intervention, it is important to highlight that the length and/or the number of sessions were fairly different, with shorter interventions being implemented in a single session (9/33; 27.3%) and the longest over a year-long course. In addition, most of the studies (23/33; 69.7%) mentioned a collaborative format promoting peer learning with groups ranging from 2—discussion group mate and “think-pair-share” (32, 42)—to 15 students (59). Furthermore, most of the studies (29/33; 87.8%) mentioned the involvement of instructional support during the sessions by contextualizing the pedagogical practice and/or the aims of the session, providing guidelines/worksheets to provide clear and standardized instructions or providing facilitators/tutors’ guidance during the session, either by rising and/or answering questions or by moderating discussions. In addition, most studies (24/33; 72.7%) mentioned that debriefing or feedback was provided during or at the end of the interventions.

Table 3 shows the assessment tools that were identified in this review. Overall, the development of CT/CR/CJ skills and/or dispositions or students’ performance in CT/CR/CJ was mainly assessed by domain-specific non-standardized tests or rubrics (19 of 26; 73.1%), while student’s knowledge, satisfaction, or perceptions about the efficacy of the pedagogical practice to foster the development of CT/CR/CJ skills were mainly assessed through domain-specific knowledge tests, self-assessment surveys, or questionnaires (5 of 7; 71.4%).

**Table 3:** Assessment tools mentioned in the selected studies (*n* = 33).

Classification	Description of the assessment tools
Domain-specific standardized tests (*n* = 5)	Health Science Reasoning Test (HSRT) (33) Diagnostic Thinking Inventory (DTI) (39) Yoon’s Critical Thinking Disposition Instrument (YCTDI) (31) Observed Structured Clinical Examinations (OSCE) (29) Critical Thinking Disposition Assessment (CTDA) (32)
General standardized tests (*n* = 2)	California Critical Thinking Skills Test (CCTST) (14)
	The Ennis-Weir Critical Thinking Essay Test + The California Critical Thinking Dispositions Inventory (CCTDI) (38)
Domain-specific non-standardized tests or rubrics (*n* = 19)	Test: students’ performance (52)
	Test: Key feature problem examination (KFPE) (44, 46, 53, 54)
	Test: Script Concordance Test (SCT) (43, 48, 50)
	Test: Medical CR performance assessed with three different knowledge tests (47, 56)
	Test: CR Test (10 problem clinical cases) (55)
	Test: Multiple choice questions to assess knowledge and CR (42, 51)
	Rubric: Critical Thinking Skills Ranking Instrument (CTSRI) (35)
	Rubric: Dual-mapping scores assessed based on a set predefined rubric (40)
	Rubric: Students performance assessed based on a matrix to measure CR skills Isaza-Restrepo et al. (49)
	Rubric: Students problem-solving performance with problem solving test and using a scoring rubric (41)
	Rubric: Interpretive summary, Differential diagnosis, Explanation of reasoning and Alternatives (IDEA) tool to assess CR skills in student hospital admission notes (57)
Domain-specific knowledge tests, self-assessment surveys, or questionnaires (*n* = 5)	Self-assessment surveys or questionnaires: Students’ perception questionnaire (34)
	Self-assessment surveys or questionnaires: students’ responses to a close-ended and open-ended questionnaire (30)
	Knowledge test: Students’ final grade at the end of the course + self-assessment surveys or questionnaires: students’ feedback at the end of the course – MBRU questionnaire (36)
	Self-assessment surveys or questionnaires: students’ perception of the enhancement of CT + survey questionnaire (9)
	Self-assessment surveys or questionnaires: self-perception survey + open-ended comments about the exercise (59)
Domain-specific focus group sessions (*n* = 2)	Students focus groups to assess students’ perceptions of the effectiveness of the approach (37)
	Students and faculty focus groups to compare students’ case creation experiences with traditional case-based learning sessions (28)

**Figure 2:**
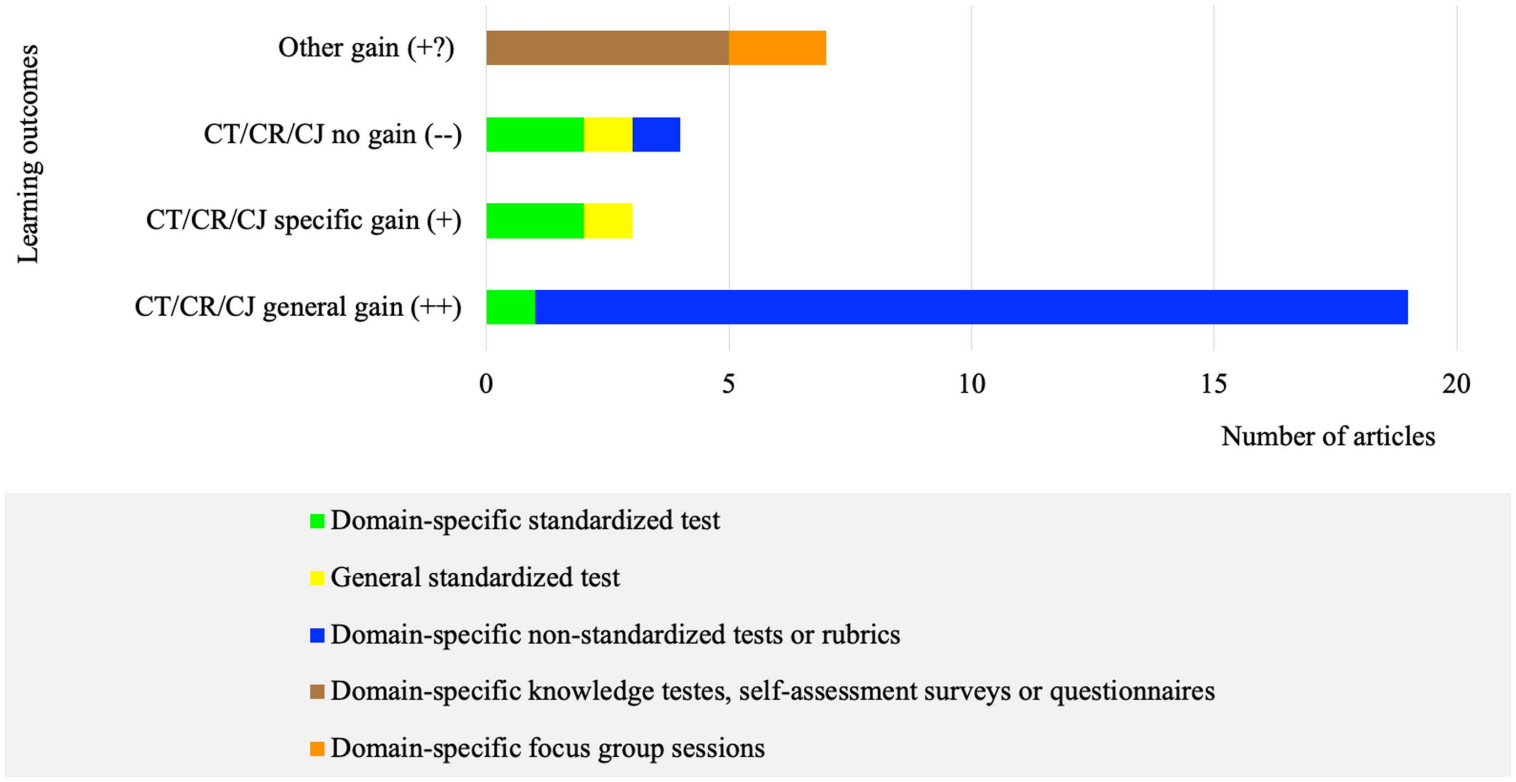
Number of articles per assessment tool regarding the learning outcomes (*n* = 33).

Of the 33 articles, 19 (57.6%) reported CT/CR/CJ general gains (++), 3 (9.1%) reported CT/CR/CJ specific gains (+), and 4 (12.1%) reported CT/CR/CJ no gains (−−). The remaining seven (21.2%) reported positive effects of the CT/CR/CJ pedagogical practices, but the gains were measured by considering improvements in knowledge or by assessing the students’ satisfaction or perceptions of the effectiveness of the intervention (Figure 2; Table 3).

Learning outcomes were also analyzed according to curricular context, subject specificity, regime, format, presence or absence of instructional support, and feedback (Tables 1, 2). Table 4 summarizes the methodological characteristics of the articles.

**Table 4:** Methodological characteristics of the selected articles (*n* = 33).

Study	Assessment	n papers	CT/CR/CJ general gain (++)	CT/CR/CJ Specific Gain (+)	CT/CR/CJ no gain (−−)	Other gains (+?)
Quantitative	Pre- and post-test	14	Watari et al (51), Moghadami et al. (43), Klein et al. (47), Ludwig et al. (53), Weidenbusch et al. (56), Si et al. (41), Kleinert et al. (52), and Raupach et al. (54)	Kim (31), Kim (32), and Taghinezhad and Riasati (38)	D'Antoni et al. (33), Bixler et al. (14), and Lee et al. (39)	–
	Only post-test	8	Nguyen et al. (35), Mutter et al. (50), Kumar et al. (42), Jost et al. (46), Middeke et al. (44), Bonifacino et al. (57), and McClintic et al. (29)	–	Schubach et al. (48)	–
Qualitative	Pre- and post-test	0	–	–	–	–
	Only post-test	2	–	–	–	Banerjee et al. (36) and Ghiam et al. (37)
Mixed	Pre- and post-test	4	Montaldo Lorca and Herskovic (55), Wu et al. (40), and Isaza-Restrepo et al. (49)	–	–	Chandrasekar et al. (28)
	Only post-test	5	Brich et al. (45)	–	–	Archila (30), Mumtaz and Latif (34), Sahoo and Mohammed (9), and Levin et al. (59)

### 3.5 Critical thinking versus clinical reasoning pedagogical practices

Of the 33 articles eligible for the review, 21 (63.6%) reported CR pedagogical practices and 12 (36.4%) reported CT pedagogical practices. No article reported interventions related to CJ pedagogical practices. Literature exposure, debate, journal club, reflective writing, dialog narrative approach, explicit CT instructions, high-fidelity patient simulation, and cognitive/visual representation (such as mind map and conceptual mapping) seem to be mainly used as CT pedagogical practices, while clinical case discussion, case creation, team-based learning, game-based learning, error-based learning, test-enhancing learning, low-fidelity simulation, and cognitive/visual representations (such as illness script, concept map, and clinical-anatomical case vignettes) seem to be mainly used as CR pedagogical practices. In addition, CT pedagogical practices were mostly applied to students attending the first (*n* = 4) and second (*n* = 3) academic years, while CR pedagogical practices, although applied in all academic years, were mostly used in the fourth year (*n* = 6), followed by third (*n* = 3), fourth plus fifth (*n* = 2), and first year (*n* = 2). Moreover, the studies that recruited students from different academic years only used CR pedagogical practices.

Regarding the multiple pedagogical practices, cognitive/visual representation and simulation were applied to the first 5 years of the curriculum. In addition, literature exposure, journal club, case creation, debate, and dialog narrative approach were implemented in the first 2 years of the curriculum, while game-based learning, team-based learning, error-based learning, and reflective writing were employed from the third to fifth years. Furthermore, domain-specific standardized tests, general standardized tests, domain-specific knowledge tests, self-assessment surveys, or questionnaires were mostly used to assess CT development, while domain-specific non-standardized tests or rubrics were mainly used to assess CR.

**Figure 3:**
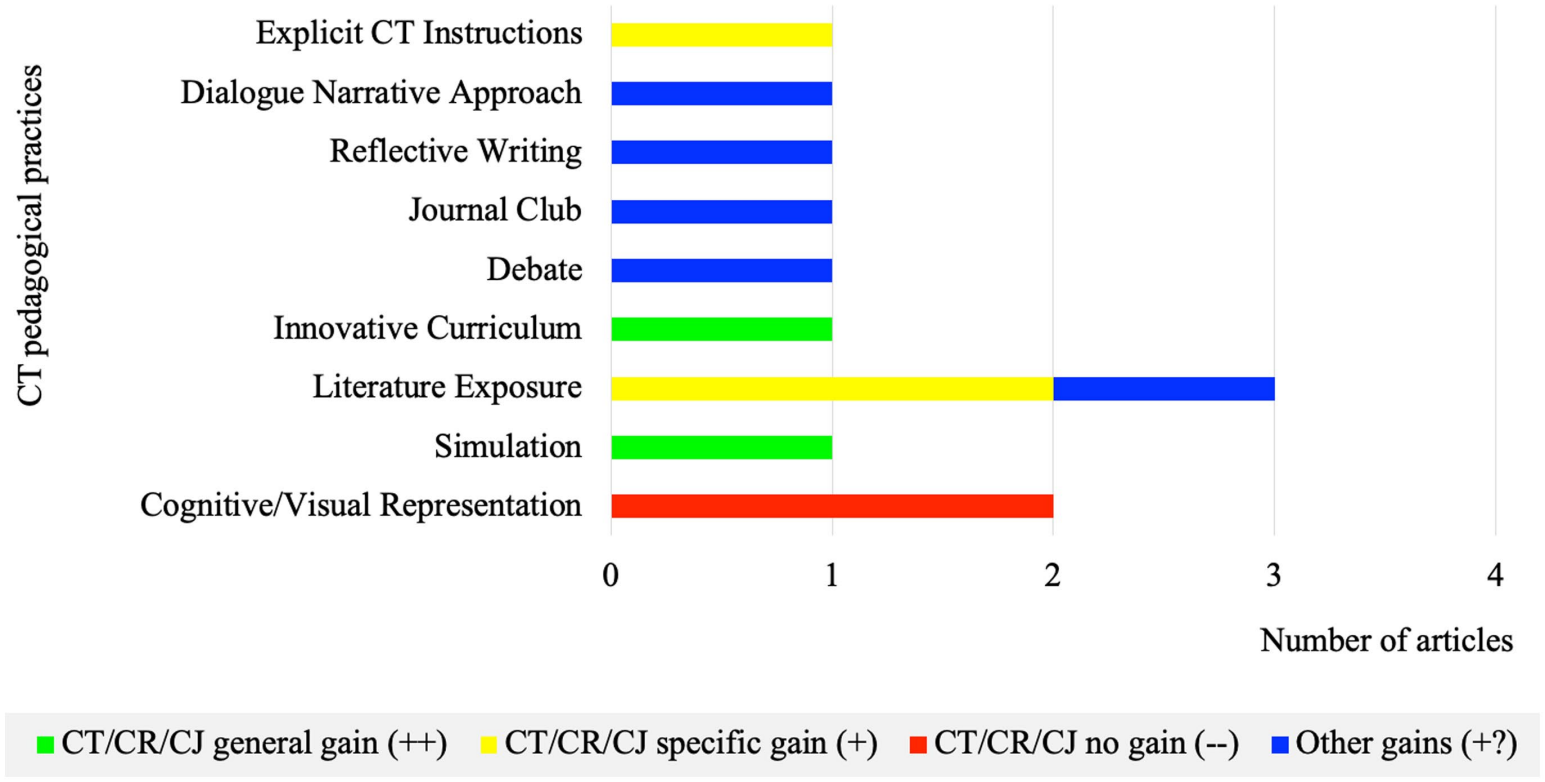
Number of articles per CT pedagogical practice regarding learning outcomes (*n* = 12).

**Figure 4:**
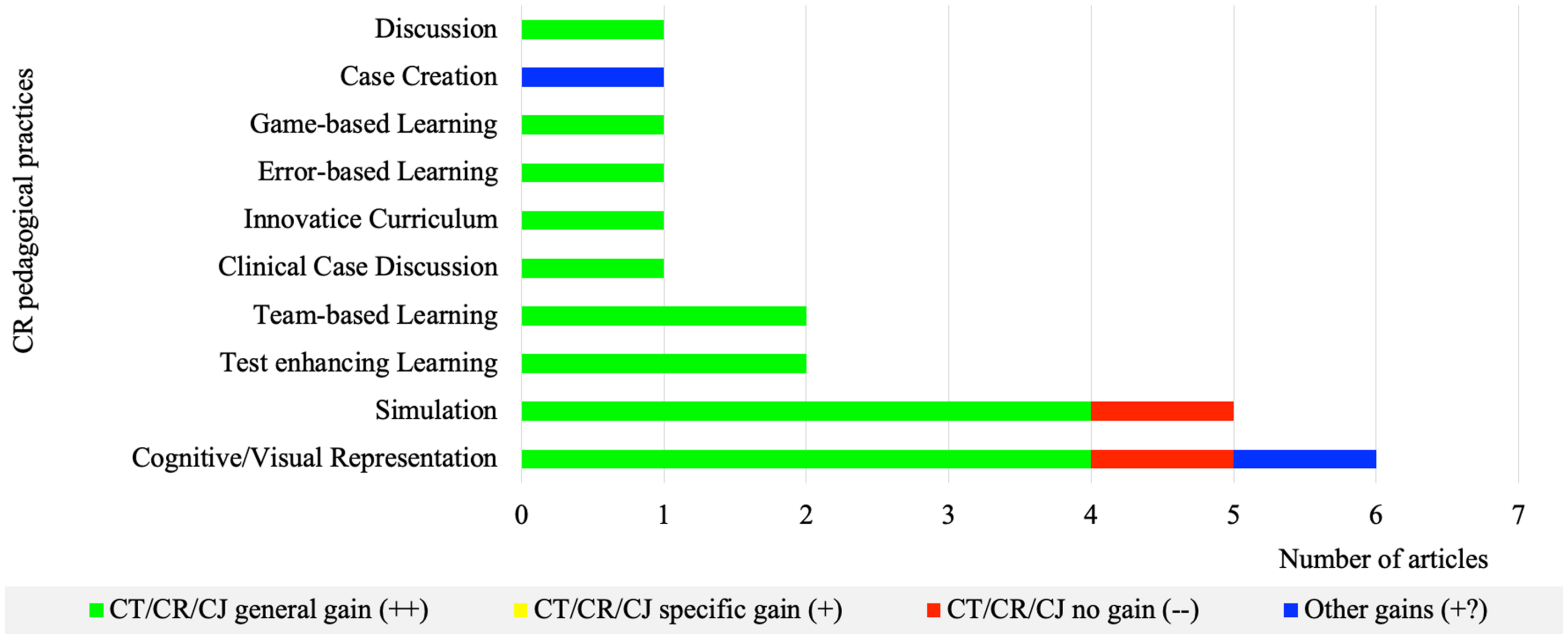
Number of articles per CR pedagogical practice regarding learning outcomes (*n* = 21).

As for the development of CT/CR/CJ skills and dispositions based on targeted outcomes, CT pedagogical practices reported mostly other gains (+?) and specific gains (+), while CR pedagogical practices reported mainly general gains (++) (Figures 3, 4).

## 4 Discussion

Due to differences in study designs and methodologies, as well as the diversity of CT/CR/CJ pedagogical practices and respective assessment tools used in the selected studies, it is challenging to identify the most effective pedagogical practices in fostering the development of CT/CR/CJ skills and/or dispositions in medical students.

Nevertheless, pedagogical practices, such as cognitive/visual representation, simulation, literature exposure, test-enhancing and team-based learning, clinical case discussion, error-based learning, game-based learning, explicit CT instructions, and the innovative curriculum approaches, seem to be effective in the development of the CT/CR/CT skills and/or dispositions as most of them reported CT/CR/CJ general gains (++). Alternatively, pedagogical practices, such as debate, journal club, reflective writing, dialog narrative approach, and case creation, seem to have a positive impact on students’ learning experience, showing improvements in student’s knowledge, satisfaction, and perception of the development of CT/CR/CJ. However, these outcomes do not directly express CT/CR/CJ since, knowledge *per se* is not enough for its development (60). In addition, the concept of CT/CR/CJ is still complex and ambiguous, leading to different perceptions among students, teachers, and experts on CT (17). Therefore, no conclusion can be inferred either from knowledge improvements or students’ perceptions of the development of CT/CR/CJ and the effective development of those skills.

When comparing CT and CR pedagogical practices, despite the differences in the medical curriculum length across countries, usually the first years are basic science-oriented while the last years are clinical training-orientated. This may be particularly interesting given that CT pedagogical practices seem to be mainly employed during the first 2 years of the curriculum, and CR pedagogical approaches are more commonly used in the last years, which may be related to the fact that CR constitutes CT application within a clinical context (61).

Most studies seem to foster CT mainly through the development of skills and dispositions. On the other hand, studies fostering CR development, in line with past evidence, were more profession-oriented and focused mainly on the development of diagnostic and decision-making skills (24, 62). In addition, the studies evaluating the effectiveness of pedagogical practices were mainly focused on fostering and assessing both CT and CR skills, whereas studies focused on dispositions were limited (*n* = 3) and only used in the context of CT (31, 32). Therefore, this can be related to how doctors conceptualize CT (i.e., in their definitions) (17). As we know, CT “requires mastery of context-specific knowledge to evaluate specific beliefs, claims, and actions” (63). Therefore, it may be useful to apply pedagogical practices that encourage the development of CT dispositions as “open-mindedness, willingness to reconsider, honesty about personal biases and persistence” (15) in the early years of the medical curriculum. As students acquire a deeper knowledge, pedagogical practices that foster CT/CR skills, more oriented to clinical decision and problem solving, could be explored. According to students’ needs and learning objectives, CT and CR pedagogical practices could be combined to achieve a more comprehensive development of skills and dispositions.

Regarding the “curricular” and “extracurricular” approaches, we have found contrasting results, with both reporting CT/CR/CJ general gains (++) (56.3% versus 58.8%) and CT/CR/CJ no gains (−−) (6.3% versus 17.6%). In agreement with previous literature, some authors believe that CT skills can be assessed regardless of the context, while others disagree (64).

Regarding the subject specificity, most studies that adopted an “immersive” followed by an “infusive” approach positively impacted the development of CT/CR/CJ skills and/or dispositions and students’ learning experiences (92.0 and 85.7%, respectively). Considering these results, and that the only study with a mixed approach reported CT/CR/CJ no gains (−−), it seems that an approach in which students are encouraged to think critically about a subject (subject-related) could be more effective, especially when they have prior knowledge on the topic. In fact, some studies show the need for a sustained specific-knowledge background to enable its application in more complex systems (60, 65). In contrast, when comparing the “immersive” and “infusive” approaches, despite the limited number of studies reporting an “infusive” approach (*n* = 7), we can suggest that the most effective are those that make these principles explicit to students. Furthermore, gains resulting from the explicit CT instructions approach corroborate this (38). Therefore, it would be interesting to incorporate CT as a specific subject in the medical curriculum, ideally during the first year.

Regarding the intervention length, some studies report that longer, progressive, and continuous interventions can lead to better outcomes, indicating that length may be an important factor in the development of CT. Although supported by a previous systematic review (24), the results herein presented are not enough to support this association.

Regarding the regime of the approach, most strategies applied the face-to-face approach, although both face-to-face and e-learning methodologies seem to positively impact the development of CT/CR/CJ.

Both individual and collaborative approaches seem to have a positive impact on the development of CT/CR. However, the learning experience can be improved by students’ engagement in discussions with each other (56). In addition, pedagogical practices seem to increase their efficacy in the following order: passive < active < constructive < interactive learning environments (66). This highlights the need to better understand the role of group interaction in the development of CT when assessing the impact of the number of students per group, both large (7 to 8 medical students) and small groups (2 medical students), and induced CT/CR/CJ general gains (++). In addition to group size, constitution seems to play a pivotal role in the effectiveness of the pedagogical practices. For instance, in heterogeneous groups, the strongest students may end up doing all the work, especially when a limited amount of time is available to perform a task. However, heterogeneity may also breed different backgrounds and perspectives on a subject, thus enriching discussion and aiding group productivity (67). In contrast, a homogeneous group will probably operate on the extremes, with the strongest groups completing their work more quickly, while the weaker groups need more time or do not complete the task by themselves (67).

Additionally, the pedagogical practice effectiveness seems to depend on the tutor and his CT proficiency (44, 68, 69). Although most studies mentioned instructional support (87.9%) or feedback (66.7%), only a minority considered their educational role and impact in fostering CT. Kim (31) highlights that activities based on interactions and feedback (e.g., group discussion and narrative comments) were more advantageous for CT development than those done individually (i.e., book reading and essay writing) (31). In agreement, some authors (28, 46, 70) suggest that the presence of an instructor through students’ guidance and prevention of misunderstandings or misconceptions can benefit both the pedagogical approach and learning experiences. Instructional support and feedback ensure that learners with learning difficulties are adequately supported in their learning process (47).

The lack of results regarding the learning outcomes may be related to differences between studies in terms of methodology and design, particularly in the intervention length, subject specificity, and assessment tools. Hence, negative outcomes might have resulted from the unfamiliarity with the approach that may have led to less proficiency (37) or from the short duration of studies with high cognitive load interventions that may compromise student adjustment to pedagogical practice and consequent performance (48).

Overall, developing CT/CR/CJ skills represents a complex learning challenge. The learner is involved in a combination of meaningful learning activities that promote both knowledge, by integrating new information into pre-existing frameworks (71), and skills such as observation, analysis, evaluation, contextualization, questioning, and, finally, reflection on a subject or a problem (72, 73). A learning environment that promotes individual growth through peer instruction, meaningful learning, and learning by doing, in which students play an active role in the learning process and receive proper feedback, is essential to ensure the efficacy of a pedagogical approach (49).

To the best of our knowledge, this is the first systematic review focused on the effectiveness of CT/CR/CJ pedagogical practices in medical education. Some limitations were identified. Although most studies were mainly focused on pedagogical practice, the presence of other approaches may have had an impact on the results. In addition, gains may also have been influenced by the educational environment, other concurrent courses, or the existence of residual or unmeasured confounders (31). Caution must also be taken with the studies describing assessment tools not previously validated for the study population (e.g., general standardized tests), as well as when a domain-specific non-standardized test or rubric was used, compromising the reproducibility of the intervention in other fields, as well as when studies relied upon a post-test assessment and/or there was no control group or other comparative intervention. Finally, no language restrictions were applied to our research strategy, but the search terms we used were in English. Given that most of the non-English language journals translate the abstract into English, we suggest that the search terms used only in English did not significantly affect the search outcomes.

### 4.1 Future research

Future studies should be planned and designed detailing the context of the intervention, the subject specificity, objectives, length, regime (face-to-face vs. e-learning), format (individual vs. collaborative), the role of the facilitators, the presence or absence of feedback, and the teacher’s expertise in CT/CR/CJ. Future research should also explore how to assess the long-term impact of the interventions on CT retention over time.

## 5 Conclusion

Pedagogical practices that actively engage undergraduate medical students in the learning process are likely more effective than traditional lectures in fostering the development of CT/CR/CJ skills and/or dispositions. However, comparison between practices is not easy due to the limited number of studies and diverse methodologies and assessment tools employed. Despite these challenges, our systematic review raises important questions about the timing, length, curricular context, regime (face-to-face vs. e-learning), and format (individual vs. collaborative) of interventions that should be carefully considered to enhance the effectiveness of the pedagogical approaches. Furthermore, it acknowledges the complexity of fostering critical thinking in medical education, recognizing that there is no “one-size-fits-all solution.” Overall, we can conclude that different pedagogical practices should be used and combined throughout the curriculum considering diverse learning environments and student needs to effectively enhance CT skills and dispositions of medical students.

## Data availability statement

The original contributions presented in the study are included in the article/Supplementary material, further inquiries can be directed to the corresponding author.

## Author contributions

BA: Conceptualization, Investigation, Methodology, Writing – original draft. SG: Investigation, Methodology, Writing – review & editing. LR: Conceptualization, Supervision, Writing – review & editing, Investigation, Methodology.
