# Supplementary material for: Critical thinking pedagogical practices in medical education: a systematic review
Source: Front Med (Lausanne). 2024 Jun 14;11:1358444. doi: 10.3389/fmed.2024.1358444 (PMC11211358; doi:10.3389/fmed.2024.1358444)
Supplement: Supplementary file 1 [file Data_Sheet_1.docx]

Supplementary Material

# Supplementary Data

| Author; Year | | (D'Antoni, Zipp, Olson, & Cahill, 2010 | (Bixler, Brown, Way, Ledford, & Mahan, 2015) | (Mumtaz & Latif, 2017) | (Nguyen et al., 2017) |
| --- | --- | --- | --- | --- | --- |
| Sample  (n \| age \| gender\| year) | | n = 131  CG=24.45 y (SD=3,26); EG=24,74 y (SD=3,91)  52 % (68) females  1^st^ year | n = 33  -  -  4^th^ year | n = 182  similar age  100 % (182) females  2^nd^ year | n = 120  -  -  1^st^ year |
| Methodology  (Pedagogical approach) | **Pedagogical practice** | **Cognitive/visual representation: Mind Map (MM)** vs Standard note-taking (SNT) | **Cognitive/visual representation: Concept mapping** in small groups | **Debate (during PBL)** | **Simulation: High-fidelity patient** simulations (HFPS) – SimMan 3G (Laerdal, Norway) manikins |
|  | **Curricular context** | Extracurricular | Extracurricular | Curricular | Curricular |
|  | **Subject Specificity** | Mixed | Immersive | Immersive | Immersive |
|  | **Subject / topic** | Cacti and other succulent plants | Pediatric topics^:^ Complications of the Infant of a Diabetic Mother / Neonatal Jaundice / Total Parental Nutrition and Fluid Management / Newborn Respiratory Disorders | Areas of controversy in medicine (doctor-patient interaction, antenatal screening, genetic testing, immunization, and alcohol abuse) | Physiology - Clinical scenarios: heat exhaustion (week 1)/ congestive heart failure (week 4) / chronic obstructive pulmonary disease (week 6) / diabetic ketoacidosis (week 16) |
|  | **Length** | 1 session (205 min) | 4 sessions (1h/session) | 1 year (2 semesters) 6-7 sessions | 17 weeks (55-60 min each session) |
|  | **Interventional and/or CGs** | CG (note-taking): n=65  IG (mind map): n= 66 | - | - | - |
|  | **Regime** | Face to face | Face to face | Face to face | Face to face |
|  | **Format** | Individual | Collaborative (groups of 4 - 6 MS) | Collaborative (20 groups; 10-13 medical students were divided into a proposition half and an opposite half) | Collaborative (20 groups - 6 MS each) |
|  | **Instructional support** | Mentioned (contextualization) | Mentioned (contextualization + guiders + tutors during the session) | Mentioned (guiders) | Mentioned (contextualization + tutors during the session) |
|  | **Feedback** | Unmentioned | Unmentioned | Mentioned (at the end of the sessions) | Mentioned (at the end of the session) |
| Methodology  (Assessment tool) | **Pre intervention** | Demographic survey  **Health Science Reasoning Test (HSRT)** – assessment of CT (33 MCQ)  **Math quiz 1 –** to “blank” the minds of the subjects  **Quiz** – assessment of domain Knowledge (5 MCQ) | **California Critical Thinking Skills Test (CCTST)**  **California Critical Thinking Disposition Inventory (CCTDI),**  Multiple choice Knowledge test (MCKT) | - | - |
|  | **Post intervention** | **Math quiz 2** (10 min)  **Quiz** - assessment of domain Knowledge (10 MCQ - 10 min)  **HSRT** – assessment of CT (33 MCQ – 45 min)  **Mind Map Assessment Rubric (MMAR)** – assessment of MM depth | **CCTST**  **MCKT**  **Online survey** – a 10 question Linkert-style survey to assess the student’s opinions and evaluation of concept mapping activity  **Additional variables** (student’s USMLE step 1 score, USMLE step 2 score and, overall pediatric clerkship grade completed in the previous years) | **Students’ perception questionnaire** regarding the usefulness of debate sessions for enhancing CT skills between other skills (11 closed-ended statements assessed by a 5-point Likert scale + open-ended questions);  **A test-retest -same questionnaire** (2 week later) with 10 students to check the reliability and validity of the questionnaire. | Basic Knowledge quiz before each HFPS (10 true or false questions covering relevant physiology and microanatomy topics from the preceding week) (one per session)  **Critical Thinking Skills Rating Instrument** (CTSRI) – 5-point Likert-scale to assess group CT skills (one per session) – median video rating of all groups. |
|  |  | **Domain Specific Standardized Tests** | **General Standardized Tests** | **Domain-specific knowledge tests, self-perception surveys, or questionnaires** | **Domain-specific non-standardized tests or rubrics** |
| Results/ conclusion | | **CT/CR/CJ No gain (-)** | **CT/CR/CJ No gain (-)** | **Other gains (+?)** | **CT/CR/CJ General gain (++)** |

CG = control group; IG = interventional group; ^1^SNT is defined as any study strategy that does not rely on reorganizing information using architecture commonly seen in a concept map or mind map; MCKT-multiple-choice knowledge test; MCQ- multiple-choice-questions

| Author; Year | | (McClintic et al., 2018) | (Archila, 2018) | (Banerjee et al., 2018) | (Sahoo & Mohammed, 2018) |
| --- | --- | --- | --- | --- | --- |
| Sample  (n \| age \| gender\| year) | | n = 214  -  44 % (94) females  3^th^ year | n = 91  16–30 years, M= 18.2 years (SD = 1.99)  62 % (56) females  1^st^ year | n = 54  -  -  1^st^ year (2^nd^ semester) | n = 188  21-23 years  56 % (105) females  4^th^ year |
| Methodology  (Pedagogical approach) | **Pedagogical practice** | **Innovative Curriculum - Entrustable professional Activities^1^** | **Literature exposure: argument evaluation - drama-based CT classroom scenarios** | **Mentored Journal clubs: 6D-approach** | **Reflective writing: Collaborative Research Protocol Writing** |
|  | **Curricular context** | Curricular | Curricular | Extracurricular | Curricular |
|  | **Subject Specificity** | Immersive | Immersive | Immersive | Immersive |
|  | **Subject / topic** | Surgical clerkship | ethics, social responsibility, and scientific work | Molecular biology and principles of genetics | Ophthalmology – “Can we prevent cataract?” and “Can we prevent diabetic retinopathy?” |
|  | **Length** | 8 sessions | 1 session (60 min) | 7 presentation sessions - 15 weeks | 4 weeks |
|  | **Interventional and/or CGs** | IG (new curriculum, EPAs): n=65  CG (traditional curriculum): n=76 | - | - | - |
|  | **Regime** | Face to face | Face to face | Face to face | Face to face |
|  | **Format** | Individual + Collaborative (small groups) | Individual | Collaborative (14 groups - 3 to 4 MS) | Collaborative (Small groups) |
|  | **Instructional support** | Mentioned (contextualization, tutors’ guidance during the session) | Mentioned (guidelines) | Mentioned (contextualization + guidelines) | Mentioned (contextualization + guidelines) |
|  | **Feedback** | Mentioned (during + at the end of the session) | Unmentioned | Mentioned (at the end of each presentation) | Unmentioned |
| Methodology  (Assessment tool) | **Pre intervention** | - Pre-Short Answer exam- to test clinically relevant material in a way that required CT. | - | - | - |
|  | **Post intervention** | - NBME Shelf score - Clinical Assessments by faculty and residents - **Post-Short Answer exam** - **Objective Structured Clinical Examinations (OSCE)** – to test students’ clinical skills - Completion of the portfolio (only the IG) - Small group performance assessed by the faculty facilitator (only the IG) | - **Participants’ responses to closed- ended (quantitative data) and open-ended (qualitative data) questionnaire.** The questionnaire comprised 3 phases to (1) decide, (2) identify and, (3) evaluate arguments form the play | - Final grade of the students at the conclusion of the course - **Students feedback at the end of the course – MBRU questionnaire** | - **Students’ perceptions of the enhancement of CT skill** through research protocol writing in small groups (qualitative method) - **survey questionnaire (**Likert scale) to identify the components that had the strongest impact to strengthen the qualitative data (quantitative approach) |
|  | | **Domain-specific standardized tests** | **Domain-specific knowledge tests, self-perception surveys, or questionnaires** | **Domain-specific knowledge tests, self-perception surveys, or questionnaires** | **Domain-specific knowledge tests, self-perception surveys, or questionnaires** |
| Results/ conclusion | | **CT/CR/CJ general gains (++)** | **Other gains (+?)** | **Other gains (+?)** | **Other gains (+?)** |

CG = control group; IG = interventional group; deliberated-practice structured orientation + small group sessions + online quizzes + extensive didactics + TBL and simulation exercises + clinical portfolio

| Author; Year | | (Kim, 2019 | (Ghiam, Loftus, & Kamel-ElSayed, 2019 | (Taghinezhad & Riasati, 2020) | (Liao & Wang, 2020) |
| --- | --- | --- | --- | --- | --- |
| Sample  (n \| age \| gender\| year) | | n = 51  21.4 +- 1.52 (19-26) years  25 % (13) females  2^nd^ year | n = 100  -  -  2^nd^ year | n=140  18-25y  Both genders  - | n=82  -  -  - |
| Methodology  (Pedagogical approach) | **Pedagogical practice** | **Literature and Film exposure:** read or watch the selected material + group discussion sessions + writing a critical essay | **Dialogue narrative approach** (storytelling format + question-answer conversational style at regular intervals) - Flipped classroom | **Explicit CT instructions^1^** | **Literature exposure:** Gender perspective into Literature + **reflection + e-discussion**: |
|  | **Curricular context** | Curricular | Extracurricular | Curricular | Extracurricular |
|  | **Subject Specificity** | Immersive | Immersive | Infusive | Immersive |
|  | **Subject / topic** | Social sciences and humanities^2^ | Thyroid physiology | English writing course  parking problem people faced in a small town  CT concepts | Gender literature studies |
|  | **Length** | 15 weeks | 1 session (50 min) | 3h per week, 1 semester, 15 weeks | 15 weeks (2xs/week) + self-study and e-discussion |
|  | **Interventional and/or CGs** | - | - | IG (CT) n= 73  CG (non-CT) n= 67 | IG (read literature works with gender perspective): n= 41; CG (read literature works for pleasure): n= 41 |
|  | **Regime** | Face to face | Face to face | Face to face | E-learning |
|  | **Format** | Collaborative (5 groups - 10 - 11 students each) | Individual | Individual | Individual + Collaborative (with group mate) |
|  | **Instructional support** | Mentioned (during the session) | Mentioned (contextualization + tutors during the session) | Mentioned (tutors’ guidance during the session) | Unmentioned |
|  | **Feedback** | Mentioned (during and at the end of the session) | Mentioned (during the session) | Unmentioned | Unmentioned |
| Methodology  (Assessment tool) | **Pre intervention** | **Yoon’s Critical Thinking Disposition Instrument (YCTDI)** | _ | The Researcher-developed Essay Test (to assess students’ performance in essays writing skills).  The Ennis-Weir Critical Thinking Essay Test (to test CT abilities).  **The California Critical Thinking Dispositions Inventory (CCTDI).** | Multidimensional Gender Consciousness Questionnaire (MGCO)- Gender awareness Scale  **Critical Thinking Disposition Assessment (CTDA)** |
|  | **Post intervention** | - The quality of the agenda and students’ performance in discussion - Critical essays evaluation - Peer-assessment performance evaluation - Students’ satisfactions and perception of usefulness of the course - survey - **YCTDI** - Students’ opinions - online questionnaire | - **Students’ perceptions of the effectiveness of the approach - Focus group sessions** – 2 groups (7 students each) (a few days after the lecture) | - The Researcher-developed Essay Test - The Ennis-Weir Critical Thinking Essay Test (to test CT abilities). - **CCTDI** | - Multidimensional Gender Consciousness Questionnaire (MGCO)- Gender awareness Scale - **CTDA** |
|  | | **Domain-specific standardized Testes** | **Domain Specific focus group session(s)** | **General standardized tests** | **General Standardized Testes** |
| Results/ conclusion | | **CT/CR/CJ Specific gain (+)** | **Other gains (+?)** | **CT/CR/CJ Specific gains (+)** | **CT/CR/CJ Specific gains (+)** |

CG = control group; IG = interventional group

| Author; Year | | (Lee et al., 2010) | (Montaldo Lorca & Herskovic L, 2013) | (Wu, Wang, Johnson, & Grotzer, 2014) |
| --- | --- | --- | --- | --- |
| Sample  (n \| age \| gender\| year) | | n = 53  -  51 % (33) females  4^th^ year | n = 64  -  -  3^rd^ year | n = 29  -  66 % (19) females  Year 4 (65.5 %) Year 3 and Year 5 (34.5 %) |
| Methodology  (Pedagogical approach) | **Pedagogical practice** | **Cognitive /visual representation: problem representation + Illness script – web-based CR problems (CRPs)** | **Clinical case discussion - Prototypical clinical cases** (lectures and tutorial sessions with patient’s vs lectures and tutorial sessions with patients + discussion seminars of prototypical clinical cases). | **Cognitive/visual representation: Computer-based argument mapping + concept mapping** |
|  | **Curricular context** | Extracurricular | Curricular | Extracurricular |
|  | **Subject Specificity** | Infusive | Immersive | Immersive |
|  | **Subject / topic** | Two scenarios: (1) an elderly man with a persistent cough” and (2) “a middle-aged woman with an acute swollen and painful left leg. | Semiology and Internal Medical clerkship  cardiac and pulmonary pathology syndromes | Kidney disease (5 learning cases) |
|  | **Length** | 1 session (3h) | 6 months | 4 weeks (5h/week) |
|  | **Interventional and/or CGs** | IG (Family medicine clerkship): n=24  CG (Psychiatry clerkship): n=29 | IG (prototypical clinical cases), n=37  CG (traditional format), n=27 | - |
|  | **Regime** | Face to face | Face to face | Face to face (introduction) + e-learning (approach) |
|  | **Format** | Collaborative (small groups) | Collaborative (small groups) | Individual |
|  | **Instructional support** | Mentioned (contextualization, tutors’ guidance during the session) | Mentioned (contextualization, tutors’ guidance during the session) | Mentioned (contextualization + guidelines + tutors’ guidance during the session) |
|  | **Feedback** | Mentioned (individualized, during + at the end of the session | Mentioned (during the session) | Mentioned (during the session) |
| Methodology  (Assessment tool) | **Pre intervention** | - **Diagnostic Thinking Inventory (DTI)** (1 week before the workshop – 20 min) - The DTI has two subscales: (1) flexibility in thinking and (2) structure in memory | - **Clinical Reasoning Test** (10 problem clinical cases) | - **Pre-Knowledge test** (3 MCQs, 10 extended matching and 4 essay questions) |
|  | **Post intervention** | - **Student’s satisfaction** – 10 – item written questionnaire. For each item, students rated the statement using a Likert scale (at the end of the workshop) - **DTI** (8 weeks after the workshop) - **CRPs score** - Individual students’ performance in solving 10 CRPs: web-based CRPs (8 weeks after the workshop - 2h) to measure knowledge | - **Clinical Reasoning Test (10 problem clinical cases)** - Focus group session to assess students’ opinion of the intervention | - Post **Knowledge test** (=) - **Dual-mapping scores** (from the first and the last dual maps generated by learners) **assessed based on a set predefined rubric.** - Self-perception learning survey (adapted from the Student Assessment of their Learning Gains Instrument) - Semi-structured written interviews to collect students’ responses to two open-ended questions: 1) advantages and disadvantages of the learning system, and 2) suggestions for improvement of the learning system. |
|  | | **Domain-specific standardized tests** | **Domain-specific non-standardized tests or rubrics** | **Domain specific non-standardized tests or rubrics** |
| Results/ conclusion | | **CT/CR/CJ no gain (-)** | **CT/CR/CJ General gain (++)** | **CT/CR/CJ General gain (++)** |

CG = control group; IG = interventional group; MCQ – multiple-choice-questions; (a) providing CT explicit instruction, (b) teaching students how to make use of those techniques to synthesize, analyze, and evaluate texts, (c) presenting support materials in CT classrooms (including leaflets, models) of the instructional techniques, (d) leading Socratic discussions based on the elements and criteria suggested in the instructional techniques, (e) assigning classroom activities and giving them adequate time to practice each skill, using both oral and written techniques, and assessing students’ performance; ^2^ materials: “sapiens” by Yuval Noah Harari / “Apeumi-giri-doeryeomyeon” by Seungsuo Kim / “Hiden Figs” /“when breath becomes air” by Paul Kalanithi / “Sesangeul-bakkun-jilmundeul” by Keyongmin Kim

| Author; Year | | (Jost, Brüstle, Giesler, Rijntjes, & Brich, 2017 | (Schubach, Goos, Fabry, Vach, & Boeker, 2017 | (Brich et al., 2017) | (Middeke, Anders, Schuelper, Raupach, & Schuelper, 2018 |
| --- | --- | --- | --- | --- | --- |
| Sample  (n \| age \| gender\| year) | | n= 26  -  58 % (15) females  4^th^ (18 students) and 5^th^ year (8 students) | n = 56  between 22-30 years old  67 % (38) females  4^th^ and 5^th^ year | n= 122  -  57,4 % (70) females,  3^rd^ (n=92) and 4^th^ (n=30) years | n=112  26,2 +/- 3,7  56,3 % (63) females  5^th^ Year |
| Methodology  (Pedagogical approach) | **Pedagogical practice** | **Team base learning (TBL)** vs non-Team base learning (non-TBL) | **Simulation: Virtual Patients (VPs) + key feature-based instructions** on multiple short cases (“key feature arm”) **vs VPs + systematic instruction** on few long cases (“systematic arm”) | **Team-based Learning** (symptom-oriented small-group seminars or sTBL units) | **Game-based learning: Serious Game (playing EMERGE)** vs small-group PBL |
|  | **Curricular context** | Extracurricular | Curricular | Curricular | Curricular |
|  | **Subject Specificity** | Immersive | Immersive | Infusive | Immersive |
|  | **Subject / topic** | Neurology topics: “vertigo”, “back pain”, “first epileptic seizure” and “acute altered mental status” | Acute abdomen  Gastrointestinal bleeding | Neurology (vertigo, acute back pain, first epileptic seizure, and acute altered mental status (AMS)) | internal medicine (cardiology/pulmology, nephrology/rheumatology, gastroenterology/endocrinology, and haematology/oncology) |
|  | **Length** | 4 sessions (90 min/session) | 3 sessions (90 min/session) 2 weeks | 2 weeks | 10 sessions (90 min/session) 6 weeks |
|  | **Interventional and/or CGs** | CG (non-TBL-class): n= 15  IG (supplementary TBL-class): n= 11 | GI_1_: (“key feature arm”): n = 30  GI_2_: (“systematic arm”): n = 26 | IG1 (sTBL + seminars), n= 122  IG2 (seminars + sTBL), n= 122 | IG_1_ (EMERGE): n=78  IG_2_ (small-group PBL): n=34 |
|  | **Regime** | Face to face | e-learning | Face to face | Face to face |
|  | **Format** | CG: individual  IG: Collaborative (groups of 5 - 7 students) | Individual work + small group discussion (2-3 MS per group) + group discussion moderated by the teacher (12 MS) | IG: Collaborative (5-7 students); CG: Collaborative (small groups) | IG_1_: individual  IG_2_: collaborative (6-8 students) |
|  | **Instructional support** | Mentioned (contextualization + guidelines + tutors’ guidance) | Mentioned (guidelines) | Mentioned (contextualization, tutors’ guidance during the session) | IG1 – Mentioned (tutors’ guidance)  IG2 - Mentioned (guidelines + tutors guidance) |
|  | **Feedback** | Mentioned (during + at the end of the session) | Mentioned | Mentioned (during and at the end of the session) | Mentioned (individualized during the session) |
| Methodology  (Assessment tool) | **Pre intervention** | - | - | - | - |
|  | **Post intervention** | - **Key feature problems examination (KFPE) to** assess CR skills (47 key features, 22 in short menu question format and 25 in long menu format) - **Multiple choice questions examination** (MCQE) to assess factual and conceptual knowledge (40 Type-A MCQs with five options each) | - **Script Concordance Test (SCT)** (18 cases/47 single questions on acute abdomen and 6 cases/12 single questions on gastrointestinal hemorrhage) to measure CR - **Course Evaluation Questionnaire** (CEQ) to assess students’ motivation and perceived efficacy of the instructional approach (using a 5-points Likert scale) - **MCQs test on factual knowledge** in visceral surgery at the end of the semester. | - multiple-choice question examination (MCQE), - **Key feature problem examination (KFPE)** - Students perceptions of both interventions – written evaluation based on the Trier Inventory for Teacher Effectiveness Evaluation | - **Key feature examination (KFE)** (6 key feature cases) - Final EMERGE session (4 patient cases - 45-minute) |
|  | | **Domain-specific non-Standardized Tests or rubrics** | **Domain-specific non-Standardized Tests or rubrics** | **Domain-specific non-standardized tests or rubrics** | **Domain-specific non-Standardized Tests or rubrics** |
| Results/ conclusion | | **CT/CR/CJ General gain (++)** | **CT/CR/CJ No gain (-)** | **CT/CR/CJ General gain (++)** | **CT/CR/CJ General gain (++)**. |

CG = control group; IG = interventional group; IG_1 –_ Intervention group 1; IG_2_ - intervention group 2; MCQ- multiple-choice-questions

| **Author; Year** | | **(Isaza-Restrepo, Gomez, Cifuentes, & Arguello, 2018)** | **(Chandrasekar et al., 2018** | **(Klein, Otto, Fischer, & Stark, 2019)** |
| --- | --- | --- | --- | --- |
| **Sample**  **(n \| age \| gender\| year)** | | n=20  -  -  1^st^ to 3^rd^ year (1^st^ to 5^th^ academic periods) | n = 10  -  -  1^st^ year | n = 84  M = 24.38, SD = 2.71  67 % (56) female,  clinical semesters: M = 8.75, SD = 2.21 |
| **Methodology**  **(Pedagogical approach)** | **Pedagogical practice** | **Simulation: Web-based Virtual patient:** low-fidelity simulator of clinical cases | **Case Creation:** “build-a-case” approach vs traditional CBL | **Error-based Learning:** learning from errors in a clinical case-based online learning environment (text vignettes) |
|  | **Curricular context** | Extracurricular | Extracurricular | Extracurricular |
|  | **Subject Specificity** | Infusive | Immersive | Immersive |
|  | **Subject / topic** | Abdomen pain of different etiology | Dilated cardiomyopathy | arterial hypertension |
|  | **Length** | 16 weeks- 2 sessions per week (2h per session) | 1 session (3h) | 1 session (no limit) in minutes: IG1: M = 41.3, SD = 19.7; IG2: M = 50.24, SD = 20.3; IG3: M = 52.9, SD = 19.4) |
|  | **Interventional and/or CGs** | - | CG (traditional CBL): n=10  IG (case creation): n=10 | IG_1_ (unsupported-example-condition): n= 29 (control group)  IG_2_ (closed-prompt-condition): n=29  IG_3_ (open-prompt-condition): n= 26 |
|  | **Regime** | face to face | Face to face | e-learning |
|  | **Format** | Collaborative (small groups) | Collaborative (2 groups - 5 MS each) | Individual |
|  | **Instructional support** | Mentioned (guidelines + tutors’ guidance) | Mentioned (contextualization + tutors’ guidance) | IG_1_- Unmentioned  IG_2_ and IG_3_ – Mentioned (guidelines) |
|  | **Feedback** | Mentioned (during the session) | Mentioned (at the end of the session) | Mentioned (at the end of the session) |
| **Methodology**  **(Assessment tool)** | **Pre intervention** | - **First clinical case- students’ performance scores assessed based on the Matrix** to measure the skills in history taking and CR skills (quantitative method). | - **Pre-self-assessment survey:** Stanford Faculty Development Program Clinical Teaching Instrument - SFDP-26 to assess the level of student–teacher interaction based on their prior traditional CBL curriculum | - **Pre-Medical reasoning performance** assessed with 3 knowledge tests: 1) conceptual knowledge test (17 MCQs); 2) strategic knowledge test (2 key feature problems); 3) conditional knowledge (1 problem solving tasks) - **Student’s self-efficacy** assessed with a 7-point Likert scale by Jerusalem and Schearzer (1999) with 10 items. |
|  | **Post intervention** | - **Last clinical case- students’ performance scores assessed based on the Matrix,** - **Students’ perceptions in a semi structured interview** (focus group), **professors’ perceptions** (field journals), and an ethnographic work from an anthropologist who attended all sessions focusing on the interaction dynamics between students, VP and professors to analyze the performance of the VP tool during the course (qualitative method). | - **Post-self-assessment surveys:** SFDP-26 to assess the level of student–teacher interaction based on their experiences during the case creation session - **Tutorial Group Effectiveness Instrument (TGEI)** to assess the group effectiveness because of this exercise and the teamwork the students experienced during the session (20 statements – 5-point Likert scale) - **Student and faculty focus group** to compare students case creation experience with traditional CBL sessions (qualitative method). | - **Cognitive Load** assessed with a 7-point Likert scale by Pass and Kalyuga (2005) with 6 items, - **Post Medical reasoning performance** assessed with 3 knowledge tests: 1) conceptual knowledge test (17 MCQs); 2) strategic knowledge test (8 key feature problems); 3) conditional knowledge (3 problem solving tasks) |
|  | | **Domain specific non-standardized tests or rubrics** | **Domain specific groups sessions** | **Domain-specific non-Standardized Tests or rubrics** |
| **Results/ conclusion** | | **CT/CR/CJ General gain (++)** | **Other gain (+?)** | **CT/CR/CJ General gain (++)** |

CG = control group; IG = interventional group; IG_1 –_ Intervention group 1; IG_2_ - Intervention group 2; IG_3_ – Intervention group 3

| Author; Year | | (Ludwig, Schuelper, Brown, Anders, & Raupach, 2018) | (Weidenbusch et al., 2019 | (Bonifacino et al., 2019) | (Si, Kong, & Lee, 2019 |
| --- | --- | --- | --- | --- | --- |
| Sample  (n \| age \| gender\| year) | | n= 93  25.8 ± 3.9 years  64.5 % (60) female  4^th^ year | n = 90  Age 20-41 years (M=23; SD=2,97)  65.5 % (59) female  1^st^ to 8^th^ semester (M=3,5; SD=1,78) | n = 67  -  -  3^rd^ year | n =95  1^st^ year (n=44: 23.1 % (15) female) Age 21-33 years (M=24, SD=2.43); 2^nd^ year (n=51: 25.2 % (24) female) Age 23-37 years (M=33, SD=2.69) |
| Methodology  (Pedagogical approach) | **Pedagogical practice** | **test-enhanced learning:** video-based key feature questions vs repeated testing with text-based KFPE | **Discussion: Clinical Case discussion** (CCD)  (Live-CCD vs Video – CCD vs Paper – cases) | **Clinical Reasoning Curriculum** six **interactive online modules** – didactic videos, simulated clinical cases and interactive prompts for open-ended MCQ; and a **case-based workshop** | **Cognitive/ visual representation: Argumentation with the concept map method during PBL** – according to Toulmin’s model of argumentation) |
|  | **Curricular context** | Extracurricular | Extracurricular | Curricular | Extracurricular |
|  | **Subject Specificity** | Immersive | Immersive | Infusive | Immersive |
|  | **Subject / topic** | cardiology, pulmonology, nephrology, rheumatology, haematology oncology | paresthesia, fever and respiratory failure, Rapidly progressive respiratory failure | diagnostic error, cognitive psychology of decision-making, specific CR skills, including use of semantic qualifiers and problem representation, and cognitive biases and heuristics | Clinical cases |
|  | **Length** | 10 weekly computer-based seminars (45 min each)  13^th^ week (exit exam), 9^th^ month (retention test) | 3 weekly - 5 sessions (90 min each), 3^rd^ week (exit exam), 5^th^ week (retention test) | 4 weeks | 3 sessions (2h each) - 3 weeks |
|  | **Interventional and/or CGs** | CG (read texts cases): n=87  IG (watch videos cases): n=87 | IG_1_: n=30; IG_2_: n=33; IG_3_: n=33 | IG: n= 34; CG: n=33 | No |
|  | **Regime** | e-learning (e-seminars) | IG_1_ (Live-CCD): face to face  IG_2_ (Video – CCD) and IG_3_ (Paper – cases): e-learning | Face to face | Face to face |
|  | **Format** | Individual | IG_1_: collaborative \| IG_2_: individual \| IG_3_: individual | Interactive modules (individual) + case-based workshop (small groups of 3-4 students + large groups) | Collaborative (7-8 students) |
|  | **Instructional support** | Unmentioned | IG_1_: mentioned (contextualization + tutors guidance during the session) \| IG_2_ and IG_3_ : unmentioned | Mentioned (contextualization + tutors’ guidance during the session) | Mentioned |
|  | **Feedback** | Unmentioned | IG_1_: mentioned (at the end of the session) \| IG_2_ and IG_3_: unmentioned | Mentioned (during the session) | Mentioned |
| Methodology  (Assessment tool) | **Pre intervention** | - **Key Feature entry examination** (4 patient cases containing 6 or 8 key features each to assess students’ performance in CR) | - **Knowledge application test/ CR performance test** (29 items - it comprised MCQs, KFPE and problem-solving tasks, addressing the conceptual (11 items), strategic (9 items) and conditional (9 items) Knowledge) (1 week before the intervention) | - - **-** | - **Individual problem-solving test** to assess individual clinical problem-solving performance. **The individual problem-solving tests used as a measure by using the scoring rubric** developed by Cho and Jonassen (2002) |
|  | **Post intervention** | - **Key Feature exist test,** at 13 weeks after 1st day of term (4 patient cases containing 6 or 8 key features each to assess students’ performance in CR) - **Key Feature retention test,** 9 months after 1st day of term (4 patient cases containing 6 or 8 key features each to assess students’ performance in CR) | - **Knowledge application** test to assess CR (at the end of the intervention) - **subjective learning outcomes** (9 items questionnaire using a 5-points Likert scale) (at the end of the intervention) - **Delayed Knowledge application test** to assess CR (2 weeks after the end of the intervention) - Student’s perception regarding the positive and negative aspects of the respective training format | - Performance on a CR Knowledge quiz (20 questions) - **Interpretive summary, Differential diagnosis, Explanation of reasoning and Alternatives (IDEA) tool to assess CR skills in student hospital admission notes**. - Student’s perception of CR concepts in their clerkship experience (5-point Likert scale). | - Concept map were evaluated by a rubric assessing their reasoning process and presentation styles - **Individual problem-solving test** to assess individual clinical problem-solving performance (after the 1^st^ PBL) - **Individual problem-solving test** to assess individual clinical problem-solving performance (after the 2^nd^ PBL) |
|  | | **Domain-specific non-Standardized Tests or rubrics** | **Domain-specific non-Standardized Tests or rubrics** | **Domain-specific non-standardized tests or rubrics** | **Domain specific non-standardized tests or rubrics** |
| Results/ conclusion | | **CT/CR/CJ General gain (++)** | **CT/CR/CJ General gain (++)** | **CT/CR/CJ General gain (++)** | **CT/CR/CJ General gain (++)** |

CG = control group; IG = interventional group; IG_1 –_ Intervention group 1; IG_2_ - Intervention group 2; IG_3_ – Intervention group 3

| Author; Year | | (Kleinert et al., 2015) | (Levin, Cennimo, Chen, & Lamba, 2016) | (Raupach et al., 2016) |
| --- | --- | --- | --- | --- |
| Sample  (n \| age \| gender\| year) | | n = 62  -  -  3^rd^ year | n = 108  -  -  2^nd^ Year | n = 87  25.0 +/- 2,9 years  58,6 % (51) females  4^th^ year |
| Methodology  (Pedagogical approach) | **Pedagogical practice** | **Simulation- Immersive Virtual Patient Simulator**: ALICE | **Cognitive/visual representation: problem representation + flipped classroom Case-base illness Script Worksheet approach** | **Test-enhanced learning:** Computer case-based learning (CBL) + augmented case presentation + Key feature questions vs repeated CBL (long case narratives) |
|  | **Curricular context** | Curricular | Curricular | Curricular |
|  | **Subject Specificity** | Immersive | Infusive | Immersive |
|  | **Subject / topic** | Esophageal cancer (3 learning cases with different tumor stages and different therapeutic options) | organ system–based unit (renal) | 3 modules: cardiology and pulmology \| nephrology and rheumatology \| haematology and oncology |
|  | **Length** | ? | 1 session (2h) | **10 sessions** - 10 weekly (45 min/session)  13 weeks (exit examination)  **9 months** (retention test) |
|  | **Interventional and/or CGs** | - | IG (Illness script worksheet format): n=59  CG (traditional question-and-answer small group format): n=121 | CG (study only cases): n=87;  IG (test cases): n=87 |
|  | **Regime** | Face to face | Face to face | e-learning (e-seminars) |
|  | **Format** | Collaborative (small groups - <5 MS) | Collaborative (groups of 15 MS) | Individual |
|  | **Instructional support** | Mentioned (guidelines + tutors’ guidance during the session) | Mentioned (contextualization + guidelines + tutors’ guidance during the session) | Unmentioned |
|  | **Feedback** | Mentioned (at the end of the session) | Mentioned (at the end of the session) | Mentioned (automatic during the session) |
| Methodology  (Assessment tool) | - **Pre intervention** | - **Students’ performance** in CR tested with pre-simulator CR files, simulator performance in case 1, and **Pre-simulator knowledge examination** (10 MCQs) | - - _____ | - **Key Feature entry examination** (4 patient cases, 30 key features to assess students’ performance in CR) |
|  | - **Post intervention** | - **Students’ performance in CR tested** with post-simulator CR files, simulator performance in case 3 and, **post-simulator knowledge examination** (10 MCQs) - **Student’s feedback** (Acceptance, effectiveness, and applicability and preexisting computer affinity were determined using a 6-point Likert scale questionnaire) - **Students’ acceptance and their opinion about the effectiveness** and applicability were determined using a (forced choice) 6-point Likert scale. | - **Self-perception survey** (using a 5-point Likert scale) - **open-ended comments about the CR exercise** | - **Key Feature exist examination,** at 13 weeks after 1st day of term (4 patient cases, 30 key features to assess students’ performance in CR) - **Key Feature retention examination,** 9 months after 1st day of term (4 patient cases, 30 key features to assess students’ performance in CR) |
|  | | **Domain specific non-standardized tests or rubrics** | **Domain-specific non-Standardized Tests or rubrics** | **Domain specific knowledge tests, self-perception survey or questionnaires** |
| Results/ conclusion | | **CT/CR/CJ General gain (++)** | **Other gains (+)** | **CT/CR/CJ General gain (++)** |

CG = control group; IG = interventional group

| Author; Year | | (Mutter et al., 2020) | (Watari, Tokuda, Owada, & Onigata, 2020) | (Kumar, Rajprasath, Priyadharshini, Murugan, & Devi, 2020) | (Moghadami, Amini, Moghadami, Dalal, & Charlin, 2021) |
| --- | --- | --- | --- | --- | --- |
| Sample  (n \| age \| gender\| year) | | n = 96  -  -  4^th^ year | n = 169  -  37 % (63) female,  4^th^ year | n =150  -  -  1^st^ year | n = 100  -  53 % (53) female  4^th^ year |
| Methodology  (Pedagogical approach) | **Pedagogical practice** | **Simulation** - **High fidelity simulation** (patient case scenario with manikin (CS-M) vs a patient case scenario without manikin (CS-NM)) | **Simulation: VPS** (®Body Interact, Portugal) | **Cognitive/visual representation: clinical-anatomical case vignettes for analyzing the clinical cases** | **Cognitive/visual representation**: Illness script + small group discussion (think aloud) + debriefing |
|  | **Curricular context** | Extracurricular | Curricular | Extracurricular | Extracurricular |
|  | **Subject Specificity** | Immersive | Infusive | Immersive | Immersive |
|  | **Subject / topic** | Chest pain | Two scenarios: (1) a 55-year-old male with altered mental status, and (2) a 65 – year-old male with acute chest pain. | Varicose veins and thyroid goiter | Cirrhosis / CHF / Nephrotic Syndrome /leg edema |
|  | **Length** | 1 session (2h) | 1 session (2h) | 2 sessions (1h each) | 2 sessions (7 h each) - 4 weeks |
|  | **Interventional and/or CGs** | IG (CS-M), n=48  CG (CS-NM), n=46 | - | IG (subjected to CR session): 150  CG: 150 | IG (with illness script): n=50  CG (without illness script): n=50 |
|  | **Regime** | Face to face | Face to face | Face to face | Face to face |
|  | **Format** | Collaborative (6 students) | Individual | Collaborative (“thin-pair-share” groups) | Individual + collaborative (180 min small group discussion + 30 min open discussion) |
|  | **Instructional support** | Mentioned (contextualization, tutors’ guidance during the session) | Mentioned | Mentioned | Mentioned (facilitator´s/ tutor´s guidance) |
|  | **Feedback** | Mentioned (during and at the end of the session) | Unmentioned | Unmentioned | Mentioned (at the end of the session) |
| Methodology (Assessment tool) | **Pre intervention** |  | - **A 20-item MCQ quiz** (10 Knowledge and **10 CR items**) to evaluate learning outcomes. | - - - | - Developed Knowledge test (10 MCQs) |
|  | **Post intervention** | - **Script concordance test (SCT**), 64-questions - Online survey to assess student’s perspectives about their engagement during the session and confidence and preparedness to apply CR skills in actual patient scenarios (Likert-scale). | - **A 20-item MCQ quiz** (10 Knowledge and 10 CR items) to evaluate learning outcomes. | - **Students’ reaction to the session** (acceptance/perceived usefulness by asking them to rate the usefulness using quantitative items (5-point Likert scale) and by snapshotting perception of students about the proceedings of CR session using nominal group technique. - **The learning gains post-test scores** (12 questions) to assess factual knowledge and CR. | - Developed Knowledge test (10 MCQs) - **Script Concordance Test (SCT)-** 10 SCTs - (4 weeks later) based on the illness scripts of these three important diseases (nephrotic syndrome, cirrhosis, and congestive heart failure) - Satisfaction questionnaire (10 items) (Likert scale) (after cross over) |
|  | | **Domain-specific non-standardized tests or rubrics** | **Domain specific non-standardized tests or rubrics** | **Domain specific non-standardized tests or rubrics** | **Domain-specific non-Standardized Tests or rubrics** |
| Results/ conclusion | | **CT/CR/CJ General gains (++)** A statistically significant mean difference on test performance between the two groups was found with the manikin group achieving higher SCT scores. | **CT/CR/CJ General gains (++)** Participants showed significant increases in average total post-test scores, both on Knowledge items and CR items. | **CT/CR/CJ General gains (++)** Mean scores in the post-test were significantly higher in the IG compared to CG. A total of 66 % students felt, diagnostic skills and lateral thinking abilities were improved, and it helped in developing problem-solving abilities for 67 % students. | **CT/CR/CJ General gain (++)** On The SCT, the mean score for the IG was significantly higher than the CG. Learner satisfaction data indicates that the intervention was well-received by students. |

CG = control group; IG = interventional group

**2. Supplementary Information - Table.** The results of Quality Assessment

| Study | | Summary score |
| --- | --- | --- |
| Qualitative studies | (Ghiam, Loftus, & Kamel-ElSayed, 2019) | 0,95 |
| Quantitative studies | (D'Antoni, Zipp, Olson, & Cahill, 2010) | 0,71 |
|  | (Bixler, Brown, Way, Ledford, & Mahan, 2015) | 0,77 |
|  | (Nguyen et al., 2017) | 0,85 |
|  | (McClintic et al., 2018) | 0,75 |
|  | (Kim, 2019) | 0,73 |
|  | (Taghinezhad e Riasiti, 2020) | 0,62 |
|  | (Liao & Wang, 2020) | 0,68 |
|  | (Lee et al., 2010) | 0,75 |
|  | (Kleinert et al., 2015) | 0,64 |
|  | (Raupach et al., 2016) | 0,82 |
|  | (Jost, Brüstle, Giesler, Rijntjes, & Brich, 2017) | 0,77 |
|  | (Schubach, Goos, Fabry, Vach, & Boeker, 2017) | 0,83 |
|  | (Brich et al., 2017) | 0,82 |
|  | (Middeke, Anders, Schuelper, Raupach, & Schuelper, 2018) | 0,71 |
|  | (Klein, Otto, Fischer, & Stark, 2019) | 0,86 |
|  | (Ludwig, Schuelper, Brown, Anders, & Raupach, 2018) | 0,75 |
|  | (Weidenbusch et al., 2019) | 0,71 |
|  | (Bonifacino 2018) | 0,73 |
|  | (Si, Kong, & Lee, 2019) | 0,77 |
|  | (Mutter et al, 2020) | 0,77 |
|  | (Watari, Tokuda, Owada, & Onigata, 2020) | 0,82 |
|  | (Moghadami, Amini, Moghadami, Dalal, & Charlin, 2021) | 0,75 |
| Mixed studies | (Mumtaz & Latif, 2017) | 0,72 |
|  | (Archila, 2018) | 0,85 |
|  | (Banerjee et al., 2018) | 0,65 |
|  | (Sahoo & Mohammed, 2018) | 0,74 |
|  | (Montaldo Lorca & Herskovic L, 2013) | 0,69 |
|  | (Wu, Wang, Johnson, & Grotzer, 2014) | 0,75 |
|  | (Levin, Cennimo, Chen, & Lamba, 2016) | 0,66 |
|  | (Isaza-Restrepo, Gomez, Cifuentes, & Arguello, 2018) | 0,65 |
|  | (Chandrasekar et al., 2018) | 0,77 |
|  | (Kumar, Rajprasath, Priyadharshini, Murugan, & Devi, 2020) | 0,67 |

# 3. Prisma Checklist

| **Section and Topic** | **Item #** | **Checklist item** | **Location where item is reported** |
| --- | --- | --- | --- |
| **TITLE** | | |  |
| Title | 1 | Identify the report as a systematic review. | 1 |
| **ABSTRACT** | | |  |
| Abstract | 2 | See the PRISMA 2020 for Abstracts checklist. | 1 |
| **INTRODUCTION** | | |  |
| Rationale | 3 | Describe the rationale for the review in the context of existing knowledge. | 1-2 |
| Objectives | 4 | Provide an explicit statement of the objective(s) or question(s) the review addresses. | 2 |
| **METHODS** | | |  |
| Eligibility criteria | 5 | Specify the inclusion and exclusion criteria for the review and how studies were grouped for the syntheses. | 3 |
| Information sources | 6 | Specify all databases, registers, websites, organisations, reference lists and other sources searched or consulted to identify studies. Specify the date when each source was last searched or consulted. | 2-3 |
| Search strategy | 7 | Present the full search strategies for all databases, registers and websites, including any filters and limits used. | 2-3 |
| Selection process | 8 | Specify the methods used to decide whether a study met the inclusion criteria of the review, including how many reviewers screened each record and each report retrieved, whether they worked independently, and if applicable, details of automation tools used in the process. | 3 |
| Data collection process | 9 | Specify the methods used to collect data from reports, including how many reviewers collected data from each report, whether they worked independently, any processes for obtaining or confirming data from study investigators, and if applicable, details of automation tools used in the process. | 3-6 |
| Data items | 10a | List and define all outcomes for which data were sought. Specify whether all results that were compatible with each outcome domain in each study were sought (e.g. for all measures, time points, analyses), and if not, the methods used to decide which results to collect. | n/a |
|  | 10b | List and define all other variables for which data were sought (e.g. participant and intervention characteristics, funding sources). Describe any assumptions made about any missing or unclear information. | n/a |
| Study risk of bias assessment | 11 | Specify the methods used to assess risk of bias in the included studies, including details of the tool(s) used, how many reviewers assessed each study and whether they worked independently, and if applicable, details of automation tools used in the process. | 4 |
| Effect measures | 12 | Specify for each outcome the effect measure(s) (e.g. risk ratio, mean difference) used in the synthesis or presentation of results. | n/a |
| Synthesis methods | 13a | Describe the processes used to decide which studies were eligible for each synthesis (e.g. tabulating the study intervention characteristics and comparing against the planned groups for each synthesis (item #5)). | n/a |
|  | 13b | Describe any methods required to prepare the data for presentation or synthesis, such as handling of missing summary statistics, or data conversions. | n/a |
|  | 13c | Describe any methods used to tabulate or visually display results of individual studies and syntheses. | n/a |
|  | 13d | Describe any methods used to synthesize results and provide a rationale for the choice(s). If meta-analysis was performed, describe the model(s), method(s) to identify the presence and extent of statistical heterogeneity, and software package(s) used. | n/a |
|  | 13e | Describe any methods used to explore possible causes of heterogeneity among study results (e.g. subgroup analysis, meta-regression). | n/a |
|  | 13f | Describe any sensitivity analyses conducted to assess robustness of the synthesized results. | n/a |
| Reporting bias assessment | 14 | Describe any methods used to assess risk of bias due to missing results in a synthesis (arising from reporting biases). | n/a |
| Certainty assessment | 15 | Describe any methods used to assess certainty (or confidence) in the body of evidence for an outcome. | n/a |
| **RESULTS** | | |  |
| Study selection | 16a | Describe the results of the search and selection process, from the number of records identified in the search to the number of studies included in the review, ideally using a flow diagram. | 6 |
|  | 16b | Cite studies that might appear to meet the inclusion criteria, but which were excluded, and explain why they were excluded. | n/a |
| Study characteristics | 17 | Cite each included study and present its characteristics. | SI |
| Risk of bias in studies | 18 | Present assessments of risk of bias for each included study. | n/a |
| Results of individual studies | 19 | For all outcomes, present, for each study: (a) summary statistics for each group (where appropriate) and (b) an effect estimate and its precision (e.g. confidence/credible interval), ideally using structured tables or plots. | 6-10 |
| Results of syntheses | 20a | For each synthesis, briefly summarise the characteristics and risk of bias among contributing studies. | n/a |
|  | 20b | Present results of all statistical syntheses conducted. If meta-analysis was done, present for each the summary estimate and its precision (e.g. confidence/credible interval) and measures of statistical heterogeneity. If comparing groups, describe the direction of the effect. | n/a |
|  | 20c | Present results of all investigations of possible causes of heterogeneity among study results. | n/a |
|  | 20d | Present results of all sensitivity analyses conducted to assess the robustness of the synthesized results. | n/a |
| Reporting biases | 21 | Present assessments of risk of bias due to missing results (arising from reporting biases) for each synthesis assessed. | n/a |
| Certainty of evidence | 22 | Present assessments of certainty (or confidence) in the body of evidence for each outcome assessed. | n/a |
| **DISCUSSION** | | |  |
| Discussion | 23a | Provide a general interpretation of the results in the context of other evidence. | 10-11 |
|  | 23b | Discuss any limitations of the evidence included in the review. | 11-12 |
|  | 23c | Discuss any limitations of the review processes used. | 11-12 |
|  | 23d | Discuss implications of the results for practice, policy, and future research. | 12 |
| **OTHER INFORMATION** | | |  |
| Registration and protocol | 24a | Provide registration information for the review, including register name and registration number, or state that the review was not registered. | 4 |
|  | 24b | Indicate where the review protocol can be accessed, or state that a protocol was not prepared. | 2 |
|  | 24c | Describe and explain any amendments to information provided at registration or in the protocol. | n/a |
| Support | 25 | Describe sources of financial or non-financial support for the review, and the role of the funders or sponsors in the review. | n/a |
| Competing interests | 26 | Declare any competing interests of review authors. | n/a |
| Availability of data, code and other materials | 27 | Report which of the following are publicly available and where they can be found: template data collection forms; data extracted from included studies; data used for all analyses; analytic code; any other materials used in the review. | n/a |

*From:*  Page MJ, McKenzie JE, Bossuyt PM, Boutron I, Hoffmann TC, Mulrow CD, et al. The PRISMA 2020 statement: an updated guideline for reporting systematic reviews. BMJ 2021;372:n71. doi: 10.1136/bmj.n71

For more information, visit: <http://www.prisma-statement.org/>
